# Meta-Analysis and Evaluation by Insect-Mediated Baiting Reveal Different Patterns of Hypocrealean Entomopathogenic Fungi in the Soils From Two Regions of China

**DOI:** 10.3389/fmicb.2020.01133

**Published:** 2020-06-12

**Authors:** Abolfazl Masoudi, Min Wang, Xiaoli Zhang, Can Wang, Zhaoxi Qiu, Wenying Wang, Hui Wang, Jingze Liu

**Affiliations:** Hebei Key Laboratory of Animal Physiology, Biochemistry and Molecular Biology, College of Life Sciences, Hebei Normal University, Shijiazhuang, China

**Keywords:** high-throughput sequencing, entomopathogenic soil fungi, baiting method, *Metarhizium*, *Beauveria*, mating type

## Abstract

A survey was carried out on forest soils and grassland soils from Hebei and Sichuan provinces using *Tenebrio molitor* larvae as a bait, and high-throughput DNA sequencing (HTS) of the fungal internal transcribed spacer-2 ribosomal DNA was used to monitor the natural distribution of three leading hypocrealean families of insect fungal pathogens (*Clavicipitaceae*, *Cordycipitaceae*, and *Ophiocordycipitaceae*). The occurrence of insect fungal pathogens in soil samples from 98 different sites was compared. The use of insect bait indicated that entomopathogenic fungi of the genus *Metarhizium* were predominant, followed by *Beauveria* and *Isaria*. Molecular characterization using the Mz_FG543 intergenic region revealed that the *Metarhizium* species pool was phylogenetically composed of three closely related species as follows; *Metarhizium pingshaense* (*n* = 74), *Metarhizium robertsii* (*n* = 51), and *Metarhizium brunneum* (*n* = 26), as well as one isolate which clustered with *Metarhizium flavoviride*. Nine potentially new phylogenetic species were delimited within the *M. flavoviride* species complex by sequencing of the 5′ elongation factor-1 alpha region locus. The *Beauveria* (*n* = 64) and *Isaria* (*n* = 5) isolates were characterized *via* sequence analyses of the Bloc region. An intergenic spacer phylogeny of the *Beauveria* isolate assemblage revealed the phylogenetic species within the *Beauveria bassiana* clade. Interestingly, the individuals of *M. pingshaense* (*n* = 18) and *M. brunneum* (*n* = 12) exhibited the presence of both mating types in Sichuan Province. Similarly, for the *Beauveria* isolates, reproductive mode assays demonstrated that all four *B. bassiana* subclades possessed bipolar outcrossing mating systems. Of these, 19 isolates contained two mating types, and the rest were fixed for single mating types, revealing opportunities for intra-lineage heterothallic mating. The HTS results showed a significantly higher occurrence of the *Clavicipitaceae* family and the *Metarhizium* genus in the soil samples. The Venn diagram showed *Metarhizium anisopliae* (*senso lato*), *Isaria farinose*, and *B. bassiana* as frequently abundant fungal pathogen operational taxonomic units (core) across sampling sites, while the baiting method showed that the genus of *Isaria* was isolated locally. The Mantel test verified that community dissimilarity increased significantly with geographical distance, suggesting that geographical coordinates are possible factors that influence the insect fungal pathogen community composition in the studied sites. This study is the first to highlight the usefulness of utilizing soil baiting and deep sequencing to investigate the population dynamics of entomopathogens in soil.

## Introduction

Entomopathogenic fungi (EFs) are a group of fungi that are pathogens of insect species. They include a broad array of diverse fungal species based on morphological characterization, phylogenetic evaluation, and ecological distribution. EFs can be found in five of the eight fungal phyla (Araújo and Hughes, 2016). Ascomycota, with approximately 64,000 known species, forms the largest group in Kingdom Fungi (Kirk et al., 2008). Its species (mostly Hypocreales) infect 13 orders of insects, the most globally diverse and widespread animals (Boomsma et al., 2014; Araújo and Hughes, 2016). The Hypocreales fungi encompass important genera of soilborne fungal pathogen taxa, and the most commonly studied genera of invertebrate pathogens are distributed among three families: *Clavicipitaceae* (*e*.*g*., *Metarhizium* and *Pochonia*), *Cordycipitaceae* (*e*.*g*., *Cordyceps*, *Beauveria*, and *Isaria*), and *Ophiocordycipitaceae* (*e*.*g*., *Ophiocordyceps* and *Tolypocladium*, formerly *Elaphocordyceps*) (Kepler et al., 2013). Communities of EFs exist in many different environments (Kepler et al., 2015; Keyser et al., 2015), and an explicit and comprehensive explanation of their structure is beneficial to address fundamental biological questions of utilizing them as microbial control agents (Behie and Bidochka, 2013). The most widely used approach for the isolation of EFs in soils is baiting with susceptible insects (Zimmermann, 1986). The wax moth larvae, *Galleria mellonella* (Lepidoptera: Pyralidae) (Vänninen, 1996; Chandler et al., 1997; Bidochka et al., 1998; Klingen et al., 2002; Quesada-Moraga et al., 2007; Sun et al., 2008; Goble et al., 2010; Meyling et al., 2011; Hernández-Domínguez and Guzmán-Franco, 2017; Inglis et al., 2019), and mealworm, *Tenebrio molitor* (Coleoptera: Tenebrionidae) (Meyling and Eilenberg, 2006; Steinwender et al., 2014; Keyser et al., 2015; Korosi et al., 2019), are the most used bait species for the general sampling of fungal entomopathogens. The outcomes of the above-mentioned surveys of naturally occurring taxa of insect-associated fungi in soil habitats are presented in Table 1. Although most studies are from subtropical or temperate areas (Hajek and Shapiro-Ilan, 2018), baiting with insects has been applied to obtain hypocrealean insect pathogens from soil samples in the tropical regions (Rezende et al., 2015) and the Arctic (Meyling et al., 2012). If part of the pathogen population is temporarily non-infective and/or non-active against the insect host at the time that the baiting occurs, the frequency of pathogens can be underestimated by this method. This issue is raised as one of the apparent biases of this detection technique (Hajek and Shapiro-Ilan, 2018). In contrast, Vega and Kaya (2012) determined this point as the main advantage of baiting on soil samples because the insect baiting method selectively isolates those pathogens which are biologically active. Some fungal species contain a narrow host range. For instance, some *Metarhizium* species such as *Metarhizium album* and *Metarhizium acridum* possess narrow-host-range pathogens of arthropods, and a standard bait method cannot detect them (Wang et al., 2016). This phenomenon might be a reasonable explanation for those studies that used different host insects as bait, resulting in some fungal species that are rarely baited with common host insects (Klingen et al., 2002; Goble et al., 2010). Furthermore, a pathogen should outcompete with a variety of other organisms and/or free-living pathogens for insects. This competition may affect the outcome of the interaction between pathogens and whole insect cadavers produced per sample. Thus, baiting with insects might underestimate the distribution patterns of soilborne insect-associated pathogens in diversity studies (Vega and Kaya, 2012; Hajek and Shapiro-Ilan, 2018). Fungal pathogen distribution patterns can be affected by many environmental factors, including abiotic and biotic factors (Jaronski, 2007, 2010; Hajek and Shapiro-Ilan, 2018), and it is difficult to draw a picture at the global scale or design general predictable patterns for EF distribution in the ecosystems. On the one hand, EF natural occurrence studies rely mostly on using the baiting method to identify insect-associated fungal pathogens on soil samples. On the other hand, various molecular techniques have been incorporated in the ecological study of invertebrate pathogens and the diseases that they cause (Hajek and Shapiro-Ilan, 2018). One of these advanced techniques is high-throughput sequencing (HTS), also named next-generation sequencing. This technique is becoming the go-to avenue for scientists studying invertebrate pathogen ecology (Malacrinò et al., 2017; Hajek and Shapiro-Ilan, 2018), especially for monitoring particular fungal species in ecosystems (Hirsch et al., 2013).

**TABLE 1:** Occurrence (%) of insect fungal pathogens from studies which used the insect bait method.

Country	Fungus	Field	Natural*	Vineyard	Isolation method	References
Finland	*B. bassiana*	5.6	28.1		*G. mellonella*	Vänninen, 1996
	*M. anisopliae*	14.9	24.2			
	*I. fumosorosea*	0.5	1.7			
United Kingdom	*B. bassiana*	1	7.7		*G. mellonella*	Chandler et al., 1997
	*M. anisopliae*	1	1.3			
	*I. fumosorosea*	0	3.3			
Canada	*B. bassiana*	35	65		*G. mellonella*	Bidochka et al., 1998
	*M. anisopliae*	63	36			
	*I. fumosorosea*	N/A	N/A			
Norway	*T. cylindrosporum*	6.7 (1.4)^†^			*D. floralis*	Klingen et al., 2002
	*M. anisopliae*	5.0 (2.9)^†^			*G. mellonella*	
	*B. bassiana*	3.3 (0)^†^			*G. mellonella*	
Spain	*B. bassiana*	34	53			Quesada-Moraga et al., 2007
	*M. anisopliae*	10	4			
	*I. fumosorosea*	N/A	N/A			
China	*B. bassiana*	27.4	86.3		*G. mellonella*	Sun et al., 2008
	*M. anisopliae*	60	26.4			
	*I. fumosorosea*	15.6	37.5			
South Africa	*B. bassiana*	13	8.3		*G. mellonella*	Goble et al., 2010
	*M. anisopliae*	3.7	4.2			
	*I. fumosorosea*	N/A	N/A			
Denmark	*B. bassiana*	9.6	53.6		*G. mellonella*	Meyling et al., 2011
	*M. anisopliae*	56.5	3.1			
	*I. fumosorosea*	0	19.6			
	*I. farinosa*	1.6	10.3			
Denmark	*M. brunneum*	78.8			*T. molitor*	Steinwender et al., 2014
	*M. robertsii*	14.6				
	*M. majus*	3.3				
	*M. flavoviride*	3.3				
Denmark	*M. flavoviride*	89.39			*T. molitor*	Keyser et al., 2015
	*M. brunneum*	9.8				
	*M. majus*	0.75				
Canada	*Beauveria* spp.	32.8	45		*G. mellonella*	Inglis et al., 2019
	*M. brunneum*	37.9	18.8			
	*M. robertsii*	15.5	3.8			
	*M. guizhouense*	1.7	1.3			
	*M. flavoviride*	1.7	N/A			
Australia	*Beauveria* spp.			26	*T. molitor*	Korosi et al., 2019
	*Metarhizium* spp.			33		

*Hedgerows or forested areas. ^†^Prevalence of entomopathogenic fungi in organical and conventional soils of farmed land. B, *Beauveria*; M, *Metarhizium*; I, *Isaria*; G, *Galleria*; D, *Delia*; N/A, not applicable.

The current research aims to discover the natural occurrence and the diversity of predominant insect-associated fungal pathogens by comparing the insect-susceptible bait method (*T. molitor* larvae) with HTS, a technique that has surprisingly not been previously examined to study the diversity of hypocrealean EFs broadly. The most abundant genus obtained by the baiting method was *Metarhizium*, followed by *Beauveria*. Molecular characterization was conducted to distinguish phylogenetic subgroups within fungal isolates, which were obtained by using the baiting method. HTS was conducted so as to target the fungal entomopathogen communities of the soil to show their taxonomic profile and their relative abundance in comparison to isolates obtained through insect baiting. After that, we turn our attention on screening all obtained *Metarhizium* and *Beauveria* using diagnostic mating-type PCR assays.

## Materials and Methods

### Ethics Statement

There was not any specific authorization required for the field sampling. The collection areas were not privately owned or protected in any way. Soil sampling was conducted from forest and grassland sites and did not include plant material from imperiled or protected species.

### Study Site and Soil Sampling

**FIGURE 1:**
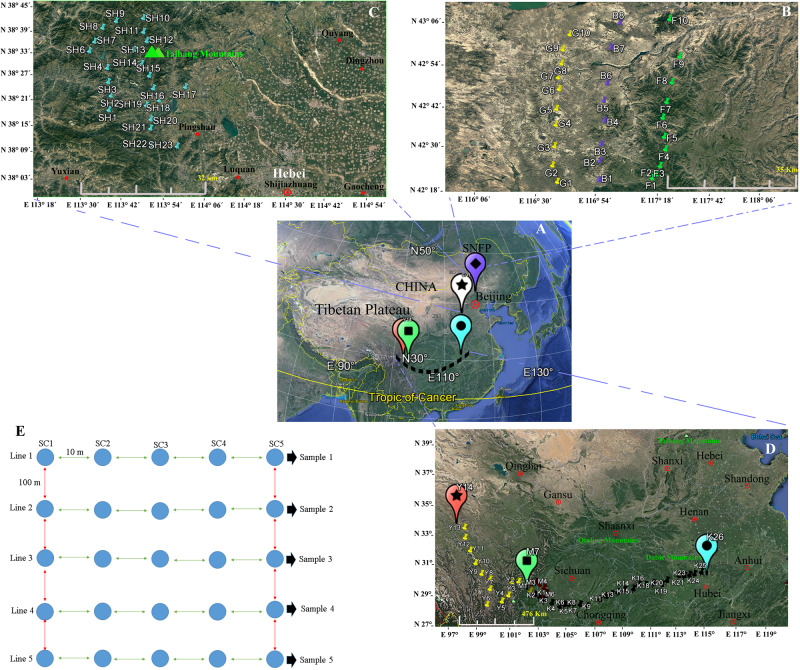
Map showing the geographical sites in China **(A)** at the Saihanba National Forest Park **(B)**, in the Taihang Mountains, and **(C)** in the area throughout Sichuan to Hubei provinces **(D)**. At each sampled locality, five lines, with a distance of 100 m between each line pair, were selected, and five soil sub-samples, within 50 m^2^ with approximately 10 m distance between each sub-sample, were collected **(E)**. A map of China and the sampling sites were generated *via* Google Earth Pro ver. 7.1.7.2600 (Google Inc.) for Windows. The sampling procedure design and the editing of the photos were performed using Xara Designer Pro X ver. 15.1.0.53605 (PANTONE^®^ LLC 2016, Xara Group Ltd.) for Windows.

We collected soil samples at the locations shown on the map (Figure 1), and the soil sampling sites were assigned coordinates *via* Global Positioning System (GPS).

Two main geographical areas were studied (Hebei and Sichuan provinces). We sampled during the peak vegetation growing season (July–August) in 2018 to target maximum microbial activity. Soil collections were conducted from Hebei Province’s Taihang Mountains in close proximity to Shijiazhuang City (23 soil samples), from now on referred to as SH (38°13′.406′′ N, 113°36′.265′′ E) and from Saihanba National Forest Park (42°14′.084′′ N, 117°08’.124′′ E) (from now on referred to as SNFP). The Taihang Mountains are located on the eastern side of China’s secondary step zone of the Bohai Bay basin and draw a physical boundary, and these also represent an important geographic barrier for the Loess Plateau and the north China meadows. The average elevation is 1,000–2,000 m. The highest elevation (2,882 m) is in the northern part of the mountain at Xiaowutai. The annual average temperature is around 10°C, and the annual rainfall is approximately 600 mm. SNFP expands across 185,000 acres of forest in Chengde. It has an average elevation of 1,400 m and two important specialties of forest and grassland as well as more than 10 plain lakes. The original ecosystem is very well preserved and maintained. It is large and the world’s biggest human-made forest park zone; its northern edge borders China’s Inner Mongolia autonomous area. The plant canopy is composed of trees, including conifer, Scots pine, birch, larch, and spruce. The sampling at SNFP was conducted at three ecotypes: forest (from now on referred to as F, with 10 samples), grassland (from now on referred to as G, with 10 samples), and the boundaries of forest and grassland (from now on referred to as B, with eight samples). Because of unfavorable weather conditions during sampling, the number of boundary samples was eventually decreased to eight (Figure 1). The second sampling was conducted in the area scattered across a gradient stretching 1,350 km west to east, in southwestern China, situated in both Sichuan and Hubei provinces, a famous biodiversity hotspot known in China and worldwide (Güneralp et al., 2015). In this area, the dominant vegetation canopy is conifer trees such as larch (*Larix* spp.), fir (*Abies* spp.), spruce (*Picea* spp.), and hemlock (*Tsuga* spp.). The climate is temperate, with an annual precipitation of 800–1,000 mm, with the most rainfall occurring between May and September, and a mean annual temperate of 6–10°C^1^.

In this region, sampling was performed to provide a comprehensive analysis of insect fungal pathogen diversity at the 47 collection sites along a 1,350-km stretch from Kangding (29°49′.516′′ N, 102°54′.276′′ E, elevation 3,500 m above sea level) to Ping Ke Xian (31°06′.962′′ N, 113°15′.18′′ E, elevation 100 m above sea level). We collected 14 samples from Kangding to Ya’an region (from now on referred to as Y) and seven samples from the Meishan region (from now on referred to as M). Sampling continued from Meishan to Ping Ke Xian (from now on referred to as K, with 26 samples). A sampling at Y and M collection sites was conducted by selecting sites that were 10 km apart from each other. The sampling sites at K location were located 50 km apart from each other (1,350 km). The leading information of the sampling sites is summarized in Supplementary Table S1. For each location, soil sampling was conducted with the identification of five lines, with the distance between each line at 100 m approximately. From each of the five lines, five soil sub-samples at 10 m distance of each site were taken from topsoil to 30–50 cm in the subsoil with a diameter of 30 cm by stepping nearly 50 m from the start point toward the end of the line. The samples were collected using clean shovels and then placing the five soil sub-samples into a plastic bowl to homogenize independently, resulting in one soil sample. As much as possible, the large roots and gravel larger than 0.25 in. were removed. The shovels were cleaned (rinsed with water and wiped dry) for each location. Then, subsamples of the homogenized material were placed in a pre-labeled zip-lock plastic bag (14 × 21 cm) separately, and the five soil samples of each site were held in a large, sealable polyethylene bag and placed in a cooler with freezer packs. The schematic soil sampling is presented in Figure 1E. In total, 490 (98 × 5) soil samples were obtained from a diverse array of sites (98 locations) representative of forest and grassland habitats. After transporting the soil samples to the lab, the samples were divided into two parts. Subsequently, 30 g of each soil sub-sample (5 × 30 g) was added to a sterile plastic bag to mix with 150 g soil manually, resulting in one composite sample, and it was immediately stored at −80°C for the conduct of a DNA extraction procedure from the soil. The second part was air-dried and ground to pass through a 2-mm-diameter sieve within 24 h to employ in the baiting with the insect.

### Soil DNA Extraction and Amplicon-Based Sequencing

Total genomic DNA was extracted in two replicates from 0.25 to 0.5 g of each composite soil sample (98 soil samples) using DNeasy^®^ PowerSoil^®^ Kit (QIAGEN, Hilden, Germany) to obtain genomic DNA free of PCR-inhibitory phenolic compounds following the manufacturer’s instructions. The two DNA extractions per soil sample were pooled before further analysis. Genomic DNA purity and integrity were determined by 1% agarose electrophoresis and nanodrop (Nanodrop 2000, Thermo Scientific, Wilmington, NC, United States). DNA was diluted to 1 ng/μl using sterile Mili-Q ddH_2_O, based on the concentration. The amplification of the DNA samples was performed using ITS3-2024F (5′-GCATCGATGAAGAACGCAGC-3′) and ITS4-2409R (5′-TCCTCCGCTTATTGATATGC-3′) primers, which is target of 350 bp of the fungal rRNA gene internal transcribed spacer-2 (ITS2) region (Orgiazzi et al., 2012). PCR amplifications were conducted in a 30-μl mixture containing 0.5 μl of bovine serum albumin (TaKaRa, Tokyo, Japan), 2.0 μl of forward and reverse primers (10 mM), 5.0 μl of the DNA sample, 7.5 μl of ddH_2_O, and 15.0 μl of Phusion^®^ High-Fidelity PCR Master Mix (New England Biolabs, Ipswich, MA, United States) with HF Buffer (New England Biolabs, Ipswich, MA, United States). PCR amplification was initiated by 98°C for 10 s, then 30 cycles of denaturation for 10 s at 98°C, annealing for 30 s at 50°C, elongation for 30 s at 72°C, and final extension for 5 min at 72°C. Subsequently, the PCR products were evaluated by examining 5 μl of the product on 2% agarose gels. The PCR assays were performed in SimpliAmp^TM^ Thermal Cycler (Life Technologies Holdings Pte., Ltd., Marsiling Industrial Estate RD3, Singapore). The samples with an intense band were purified with GeneJET ^TM^ Gel Extraction Kit (Thermo Scientific, Waltman, MA, United States). Sequencing libraries were produced using Ion Plus Fragment Library Kit 48 reactions (Thermo Scientific, South San Francisco, CA, United States) according to the manufacturer’s instructions, and index codes were added. The library quality was assessed on the Qubit 2.0 Fluorometer (Thermo Scientific, United States) and Agilent Bio-analyzer 2100 system (Agilent, Santa Clara, CA, United States). The clustering of the index-coded samples was performed on a cBot^TM^ 2 Cluster Generation System according to the manufacturer’s instructions. Having generated the cluster, the prepared libraries were sequenced on an Ion S5^TM^ XL SE400/SE600^2^ at Novogene Bioinformatics Technology Co., Ltd. (Tianjin, China), and single-end reads were generated. The raw sequences produced in the current study can be accessed through the National Center for Biotechnology (NCBI)^3^ under BioProject ID PRJNA551928, accession SUB5873947.

### Operational Taxonomic Unit Cluster and Diversity Analysis

To obtain high-quality clean reads and to improve the accuracy of the contig lengths of the resulting assemblies, quality filtering on the raw tags (splicing sequences) was carried out following particular filtering circumstances that were performed using the Cutadapt ver. 1.9.1 (Martin, 2011) with default settings^4^. The comparison between sequence reads with the reference database (UNITE database^5^) (Abarenkov et al., 2010) was performed to distinguish and to remove chimeric sequences using UCHIME algorithm (Edgar et al., 2011). The UNITE database ver. 6.0 for QIIME^TM^ ver. 1.9.1 software pipeline^6^ (Caporaso et al., 2010) was applied as a source file for operational taxonomic unit (OTU) picking and assigning the classification for the three hypocrealean fungal families, and the rest of the OTUs were deleted (Caporaso et al., 2010; Kõljalg et al., 2013). The sequence from each OTU with the highest frequency was then selected as an illustrative sequence of that OTU. The clusters that comprised two sequences, one sequence was randomly picked, and single read clusters were excluded from the data. The clusters were then taken together into a matrix with cluster absolute and relative abundance *versus* sample identities. Each OTU was taxonomically identified using UPARSE software ver. 7.0.1001 (Edgar, 2013) *via* a combined comparison with the curated database UNITE (Abarenkov et al., 2010) and with a batch BLAST search against GenBank (NCBI) (Altschul et al., 1990). The ITS2 homology for defining taxa in GenBank was fixed to at least 97–100% and e-values below e^–100^ for species, 90–97% and e^–90^ for genus level, and 80–90% and e^–80^ at the family level, corresponding to at least 90% of the sequence length (Ottosson et al., 2015).

Taxonomic data were assigned to each representative sequence using the QIIME^TM^ software after de-noising the reads using the QIIME^TM^ software (Reeder and Knight, 2010). Multi-sequence alignments were performed using MUSCLE software ver. 3.8.31 (Edgar, 2004) to examine the differences of the dominant species in different groups and finally to construct the phylogenetic relationship of different OTUs. The frequency of OTUs was normalized using a sequence standard based on the sample with the fewest sequences. Also, we visualized relative abundance and taxonomic hierarchy using Krona radial space filling (Ondov et al., 2011). The order, family, and genus ranks of each sample were selected for representation in the Krona plots. Since EFs were considered in this study, only Hypocreales order was chosen to generate these plots. The less abundant taxa are listed outside the charts along with their relative abundance. Four indices of alpha diversity, including Chao1 (Chao, 1984), Shannon (1948); Simpson (1949), and Faith’s phylogenetic diversity (Faith, 1992), were analyzed based on the complexity of species diversity *via* observed OTUs for a sample using QIIME^TM^. To evaluate the differences of samples in species complexity, beta diversity on both weighted and unweighted UniFrac (Lozupone et al., 2007) was determined by QIIME^TM^. The results were visualized in phylogenetic trees and box plots. The abundance of distinguished OTU with high frequency (>0.1%) between sample locations was delimited using *t*-test and Kruskal–Wallis test (Kruskal and Wallis, 1952). To recognize the taxa with significant differential abundance, a linear discrimination analysis effect size (LEfSe) method was computed, where different sampling locations were assigned as comparison classes. The non-parametric factorial Kruskal–Wallis sum–rank test was applied to analyze all LEfSe-identified features that were significantly different among sampling sites [*P* < 0.05, linear discriminant analysis (LDA) ≥ 4] (Segata et al., 2011). Furthermore, to evaluate whether the beta diversity of insect fungal pathogen communities was related to the coordinates of the sampling locations, we performed a distance-based redundancy analysis (dbRDA) [Legendre and Anderson (1999) in the package Vegan ver. 2.3.5 (Oksanen et al., 2013) in R ver. 3.2.1 (R Core Team, 2016)]. We used a permutational analysis of variance to test for the significance of terms in the dbRDA. The Mantel test was applied with a significance testing against 999 permutations in R using Vegan package ver. 2.3.5 (Oksanen et al., 2013). Non-metric multidimensional scaling (NMDS) was applied to visualize community differences. Analysis of similarity (ANOSIM), a non-parametric multivariate analysis, was performed to test the significance of the patterns observed by NMDS. The distance matrix for this analysis was calculated using the Bray–Curtis similarity index. ANOSIM was performed using the Vegan package. The effectiveness of sampling was evaluated with a rarefaction analysis of data subsets using the specaccum function (Oksanen et al., 2013) in R (Supplementary Figure S1).

### Insect–Fungal Pathogen Isolation

#### Fungal Isolation

The insect-susceptible bait method applied in the current study was adjusted from Zimmermann (1986) and Keyser et al. (2015). Enough ddH_2_O was added to the prepared soil samples to moisten them as described before. Approximately 200 g of soil was transferred in a polyethylene container (500 ml), omitting 15 cm of airspace at the top. Ten healthy 5–6^th^ instar larvae (1–2 cm) of *T. molitor* L. (Coleoptera: Tenebrionidae) were then transferred to each plastic container. A ventilated lid with oxygenating holes (1–2 mm in diameter) was added on top of each plastic container, and the containers were turned upside down to busy the larvae in a container. Every day the containers were rotated so that the orientation of the cups shifted, driving the *T. molitor* larvae to move within the soil substrate and enhancing the probability that they would come in touch with insect-associated fungi in the soil. Insect survival was monitored weekly; dead insects were removed, washed with ddH_2_O, and placed in a sterile Petri plate (35 × 10 mm) with a moistened filter paper to determine mycosis. We should mention that, due to the enormous number of soil samples, surface sterilization of the *T. molitor* larvae did not apply to the fungal isolation procedure (Meyling, 2007). Fungal isolations were made using a sterile toothpick from insect cadavers with mycosis and plated on PDAY media plus 0.6 g streptomycin sulfate (MP Biomedicals, Shanghai, China) and 0.5 g chloramphenicol (MP Biomedicals, Shanghai, China). After 3 weeks of growth, the colonies were evaluated morphologically and diagnosed to the genus level based on the conidial shape, phialides, and hyphae (Humber, 2012). A single-spore isolation method was applied to purify all the obtained fungal isolates using a continuous dilution of the conidia suspension of each isolate (Choi et al., 1999). We considered each infected cadaver as one isolate (Korosi et al., 2019). Finally, a small piece of agar plug of each fungal monosporic isolate was placed into a sterile 2-ml Eppendorf tube containing 20% sterilized glycerol to keep them at −80°C for further studies. The natural occurrences of the obtained fungal isolates were assessed for homogeneity among all collection sites using Pearson’s χ^2^ test in GraphPad Prism ver. 8.0.2 (GraphPad Software, Inc.)^7^.

#### Fungal Genomic DNA Extraction, PCR Amplification, and Sequencing

DNA extraction was performed by inoculating the fungal conidia (10^5^ conidia/ml) into the wells of a 96-well tissue culture plate (Thermo Fisher Scientific, United States). Each well contained 1 g yeast extract (OXOID, Basingstoke, Hampshire, United Kingdom), 0.5 ml of 1 g peptone (AOBOX, Beijing, China), and 4 g D-glucose anhydrous (Solarbio, Beijing, China) (all weights per liter). The plates were incubated at 27°C in an incubator for 72 h. The fungal hyphae were collected from the wells by centrifuging 0.5 ml of the re-suspended hyphae in a sterilized micro-centrifuge tube The fungal hyphae were lyophilized using Virtis BenchtopPro Freeze Dryer with Omnitronics^TM^ (Genevac Ltd., Ipswich, United Kingdom). Total genomic DNA for all fungal isolates was extracted by pulverizing lyophilized hyphae with lysis buffer and 0.5 g of 0.5–1-mm-diameter acid-washed, sterilized glass beads (Tiantai Jingong SiLi Glass Beads Co., Ltd., Tiantai, Zhejiang, China) by using Bullet Blender^®^ 24 Gold Tissue Homogenizer (Next Advance, Inc., NY, United States). The rest of the DNA extraction procedure was continued by using the DNeasy^®^Plant Mini Kit (QIAGEN, Hilden, Germany) following the manufacturer’s instructions. Isolated DNA was quantified by using a TU-1950 spectrophotometer (Yima Opto-electrical Technology Com., Ltd., Xi’an, China) and stored at −20°C until needed. For *Metarhizium* isolates, PCR amplification and sequencing approaches for the intergenic region, Mz_FG543igs gene, were performed (Kepler et al., 2013, 2015; Keyser et al., 2015). The master mix reagents for each PCR reaction consisted of 6.5 μl 2x Easy Taq^®^ PCR SuperMix (TransGen Biotech Co., Ltd., Beijing, China), 2.5 μl of each primer, 11.5 μl Milli-Q H_2_O, and 2 μl DNA sample. The primers used were Mz_FG543igs_1F (5′-ATT CAT TCA GAA CGC CTC CAA-3′) and MzFG543igs_4R (5′-GGT TGC GAC TCA CAA TCC ATG-3′). PCR amplification was initiated at 95°C for 2 min, then 40 cycles of denaturation for 30 s at 95°C, annealing for 30 s at 62°C, elongation for 60 s at 72°C, and a final extension for 15 min at 72°C. For phylogenetic placement of *Beauveria* and *Isaria* isolates, the partial sequence of the nuclear intergenic region Bloc was amplified by PCR using the primer pair B5.1F (5′-CGA CCC GCC AAC TAC TTT GA-3′) and B3.1R (5′-GTC TTC CAG TAC CAC TAC GCC-3′) (Rehner et al., 2011). The PCR cycle conditions for Bloc region were as follows: 94°C for 3 min as initial denaturation, followed by 35 cycles of 94°C for 30 s, 56°C for 30 s, and 72°C for 1 min, and a final extension of 72°C for 10 min. The PCR assays were performed in a Mastercycler^®^ Pro S (Eppendorf AG, Hamburg, Germany). The PCR products were visualized on 2% agarose gel to ensure strong single bands and purified using Wizard^®^ SV Gel and PCR clean-up kit (Promega Corporation, Madison, WI, United States). All the primers used for amplification were also used for sequencing. Positive amplicons were sequenced in both directions in their entirety to ensure accurate readings at both primers’ ends with the diluted PCR primers (10 pmol/μl) using MGISEQ-2000 Genetic Sequencer (BGI-Tech, Beijing, China). The sequences were edited, and the forward and reverse reads were assembled into contigs as implemented in BioEdit Sequence alignment editor ver. 7.2.5 for Windows software (Hall, 1999). All sequences were deposited at NCBI under the accession numbers listed in Supplementary Table S2.

### Phylogenetic Analyses

All the fungal sequences were typically examined for initial taxonomic diagnosis through BLAST-based (Altschul et al., 1990) similarity search in the Genbank database. To avoid inappropriate diagnosis, our molecular identification was not completely reliant on the database searches. Therefore, we used phylogenetic analyses to clarify the taxonomic placement of the obtained isolates. Additionally, sequences of known *Metarhizium* spp. and *Beauveria* spp. isolates from Rehner et al. (2011) and Kepler et al. (2014) were included in the phylogenetic analyses. In the second step, the fungal sequences were aligned to the reference sequence alignment with MAFFT ver. 7.0 (Katoh and Standley, 2013) with the default settings as implemented on CIPRES Science Gateway ver.3.3 (Miller et al., 2010)^8^. The nucleotide alignments of all datasets were used separately to infer maximum likelihood phylogenies as implemented in RAxML, using the RAxML-HPC2 on XSEDE (Stamatakis, 2014) web-server at CIPRES Portal (Miller et al., 2010). Bootstrap support was determined using rapid bootstrapping with 1,000 replicates. Bootstrap values ≥ 70 were considered as significant (Kepler et al., 2014). The GTRGAMMA model was applied for all datasets. The final dendrograms were depicted using MEGA ver. 7.0.26 for Windows software (Kumar et al., 2016). The final phylogenetic trees were edited by Adobe Illustrator CC, 2019.^9^

### PCR Diagnosis of *Beauveria* and *Metarhizium* Mating Type

All obtained *Beauveria* and *Metarhizium* isolates were screened for internal segments of MAT1-1-1 and MAT1-1-2 mating type idiomorphs using PCR. The primer sequences and the PCR cycle conditions for *Beauveria* and *Metarhizium* were used according to Meyling et al. (2009) and Kepler et al. (2015), respectively.

## Results

### HTS Data Output

#### Ion S5 XL Sequencing and Sequence Analysis

In order to delineate the composition of insect–fungal pathogen communities among different geographical locations, we conducted high-throughput Ion S5 XL paired-end sequencing. The sequencing statistics have been summarized (Supplementary Table S3). A total of 7,790,252 reads were obtained from 98 soil samples; 5,979,723 high-quality reads (49,619 ± 42,532 reads per sample), with an average length of 304 bp, were retained after length filtering as well as removal of chimeric sequences. The validated reads ranged from 73,884 (M) to 80,335 (G). The total count ranged from 2,2430,757 (SH) to 2,436,401 bp (G). The length of the average reads ranged from 302.31 to 305.307 bp. The average GC content percentage ranged from 49.46 to 50.87% (Supplementary Table S3). Finally, the reads were sorted into samples by detecting the barcodes for global fungal databases (UNITE) for the three entomopathogenic hypocrealean family genera, and the rest of the sequences were removed. Consequently, 31,713 sequence reads of insect–fungal pathogens ITS2 (20,427 sequence reads of *Clavicipitaceae*, 7,885 sequence reads of *Cordycipitaceae*, and 3,401 sequence reads of *Ophiocordycipitaceae*) were clustered into 615 OTUs. The K sampling sites were detected with the maximum number of EF OTUs (219) out of 1,681 average fungal OTUs (13.02%), and the lowest number of EF OTUs (14) was identified from the B sampling site out of 1,186 average fungal OTUs (1.18%) (Supplementary Figure S2 and Supplementary Table S4).

#### Taxonomic Composition and Abundance Analysis

**FIGURE 2:**
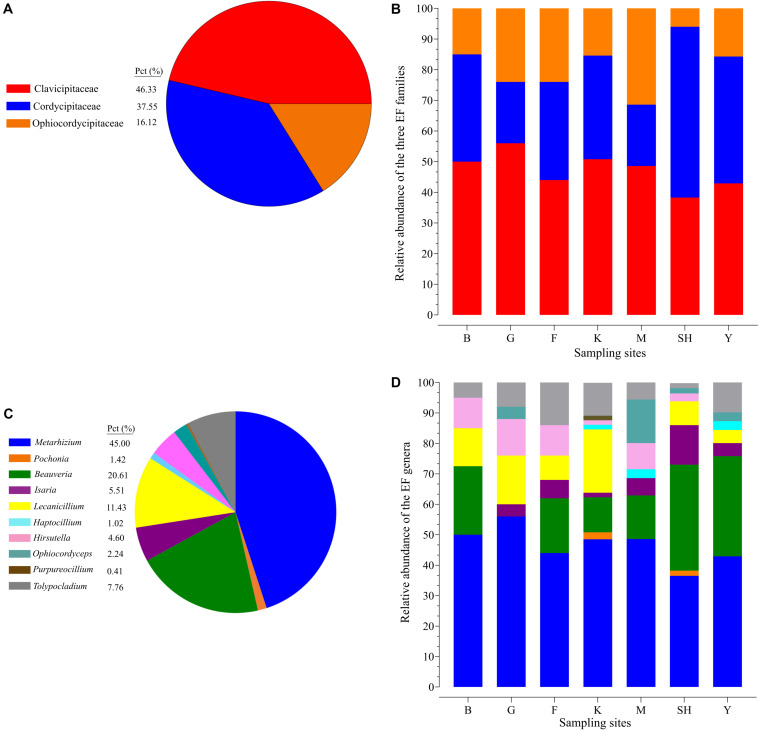
Relative taxonomic abundance of soil-inhabiting entomopathogens. The relative abundance of three hypocrealean insect-associated fungi at all the 98 collection sites **(A)** and at each sampling location separately **(B)**. Relative abundance of the top 11 entomopathogenic fungi genera at all the 98 collection sites **(C)** and at each sampling location separately **(D)**. SH (Taihang Mountains), K (Meishan to Ping Ke Xian), Y (Ya’an area), M (Meishan region), F (Saihanba National Forest Park; forest), G (Saihanba National Forest Park; grassland), and B (Saihanba National Forest Park; the boundary of forest and grassland).

The average relative abundance of EFs from the overall fungal community in terms of the sampling site revealed results in the following order Y > K > SH > M > F > G > B (0.60, 0.53, 0.41, 0.40, 0.087, 0.079, and 0.067%). The summary of the results revealed that insect–fungal pathogen sequences belonged mainly to *Clavicipitaceae* (46.33%), followed by *Cordycipitaceae* (37.55%), and *Ophiocordycipitaceae* (16.12%) (Figure 2A). The *Clavicipitaceae* family had the highest relative abundance in all the sampling locations in forest ecosystems and in grassland ecosystems except at the SH location (38.30%) (Figure 2B), and *Cordycipitaceae* (55.70%) prevailed at the SH location (Figure 2A). The most abundant EFs genus was *Metarhizium* (44.49%), followed by *Beauveria* (20.61%) and *Lecanicillium* (11.43%) (Figure 2C). All sampling locations were dominated by the genus *Metarhizium*, but its abundance followed the trend of G > B > M > K > F > Y > SH (56.0, 50, 48.6, 45.4, 44.0, 42.9, and 36.5% of total EF OTUs, respectively) (Figure 2D). The relative abundance of *Metarhizium* differed significantly between B and SH (*P* = 0.0017), between G and SH (*P* = 0.0038), and between B and Y (*P* = 0.0259). *Metarhizium* was the most predominant genus in all the sampling locations except at the SH location, in which *Beauveria* (42.01%) was found to be prevalent.

**FIGURE 3:**
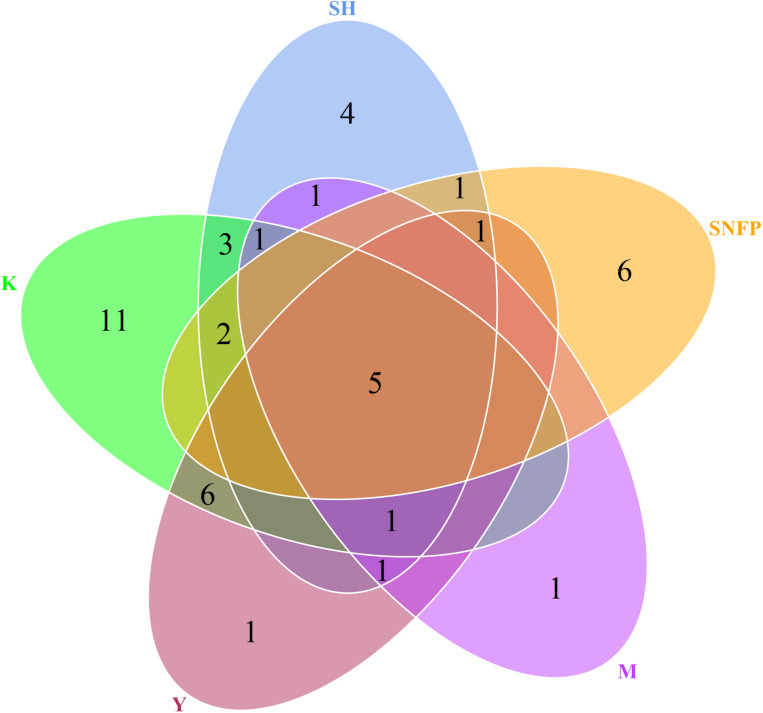
Hypocrealean entomopathogenic soil fungal diversity. Venn diagram depicting the number of shared and unique operational taxonomic unit counts among all collection sites. Zeros have not been displayed for better comprehension. SH (Taihang Mountains), SNFP (Saihanba National Forest Park), Y (Ya’an area), M (Meishan region), and K (Meishan to Ping Ke Xian).

The Venn diagram displays the shared OTU counts as well as the unique OTU counts among the collection sites. The K collection site had the highest number of unique OTUs (11), representing 8.46% of the K collection site OTUs. The lowest number of unique OTUs was displayed by the Y collection sites and the M collection sites (one). The SNFP and the SH sampling sites displayed six and four unique OTUs, respectively. Y had the highest number of shared OTUs, with K (six). Overall, only five of the total number of OTUs determined in the different samples were present in all the individual samples, and these may be considered as forming a part of the ‘core’ soil insect fungal pathogens (Figure 3). The core OTUs were identified as *Metarhizium* sp., *Beauveria* sp., *Metarhizium anisopliae*, *Beauveria bassiana*, and *Isaria farinose.*

**FIGURE 4:**
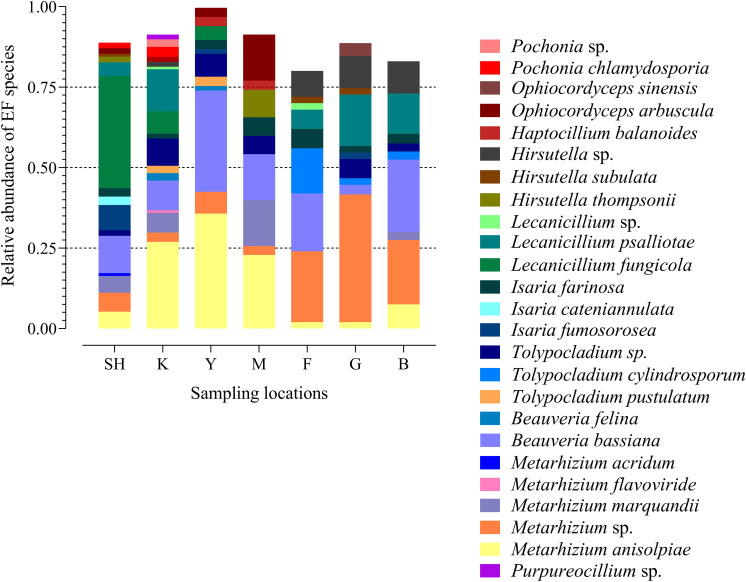
Species-level assignment of entomopathogenic fungi detected in three families (*Clavicipitaceae*, *Cordycipitaceae*, and *Ophiocordycipitaceae*) at seven collection locations (98 sampling sites) based on the frequency of operational taxonomic units. SH (Taihang Mountains), K (Meishan to Ping Ke Xian), Y (Ya’an area), M (Meishan region), F (Saihanba National Forest Park; forest), G (Saihanba National Forest Park; grassland), and B (Saihanba National Forest Park; the boundary of forest and grassland).

The frequency of 25 EF species from three fungal families in each sampling location has been shown in the species-level assignment plot (Figure 4). The most abundant EF species was *Metarhizium* sp. from the G sampling location (39.67%), while *Metarhizium flavoviride* and *Lecanicillium* spp. from the K sampling location showed the lowest abundance (0.7%). Several taxa at the species level, including *Purpureocillium* sp. (1.53%), *Pochonia* sp. (2.3%), *Ophiocordyceps sinensis* (4.1%), *Isaria cateniannulata* (2.6), *M. acridum* (0.8%), and *M. flavoviride* (0.7%), were detected in just one sampling location. Frequency analysis at the species level highlighted the presence of *Tolypocladium* sp. at all the sampling sites except at the F collection site (Figure 4).

#### Alpha Diversity

Species richness, as indicated by the Chao1 index, differed significantly between the Y and the G sampling locations (*P* = 0.0491). Maximum chao1 and minimum chao1 indices were presented by the SH (4.717) and the G (2.45) sampling sites, respectively. The Shannon index displayed a significant difference between the G and the M (*P* = 0.0356) and the K sampling locations (*P* = 0.0440). The highest values and the lowest values of the Shannon index were observed for the G (0.842) and the M (1.536) sampling sites, respectively. Moreover, the Simpson index showed a significant difference between the M and the B collection sites (*P* = 0.0349). The highest and the lowest values of the Simpson index were detected at the G (0.352) and the M (0.605) sampling sites, respectively. The Faith’s phylogenetic diversity ranged from 0.518 to (Y) 0.609 (M) (Supplementary Table S4).

#### Beta Diversity

**FIGURE 5:**
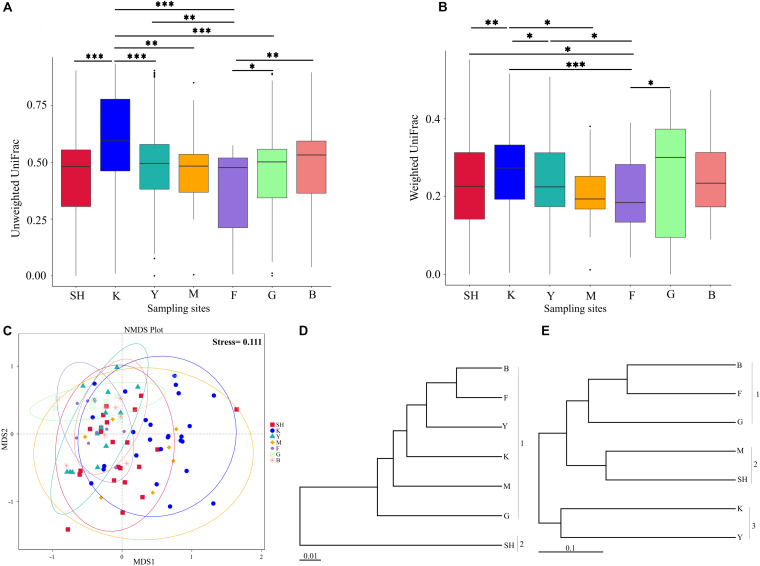
Microbial community composition of samples from different collection sites. Distance boxplots of the unweighted **(A)** and the weighted **(B)** UniFrac matrices were used to evaluate the diversity between groups. Pairwise comparison was computed for all pairs of boxplots (sampling groups) using the Wilcoxon test (***, adjusted *P* < 0.001; **, adjusted *P* < 0.01; *, adjusted *P* < 0.05.). **(C)** Non-metric multidimensional scaling plot and Bray–Curtis distances have been used, and ellipses represent 95% certainty interims around the mean centroid of each sampling location. **(D,E)** UPGMA trees based on the UniFrac analysis on the normalized weighted **(D)** and the unweighted **(E)** data. The scale bars represent the number of expected changes per site. Data represent collection sites at SH (Taihang Mountains), K (Meishan to Ping Ke Xian), Y (Ya’an area), M (Meishan region), F (Saihanba National Forest Park; forest), G (Saihanba National Forest Park; grassland), and B (Saihanba National Forest Park; the boundary of forest and grassland).

**FIGURE 6:**
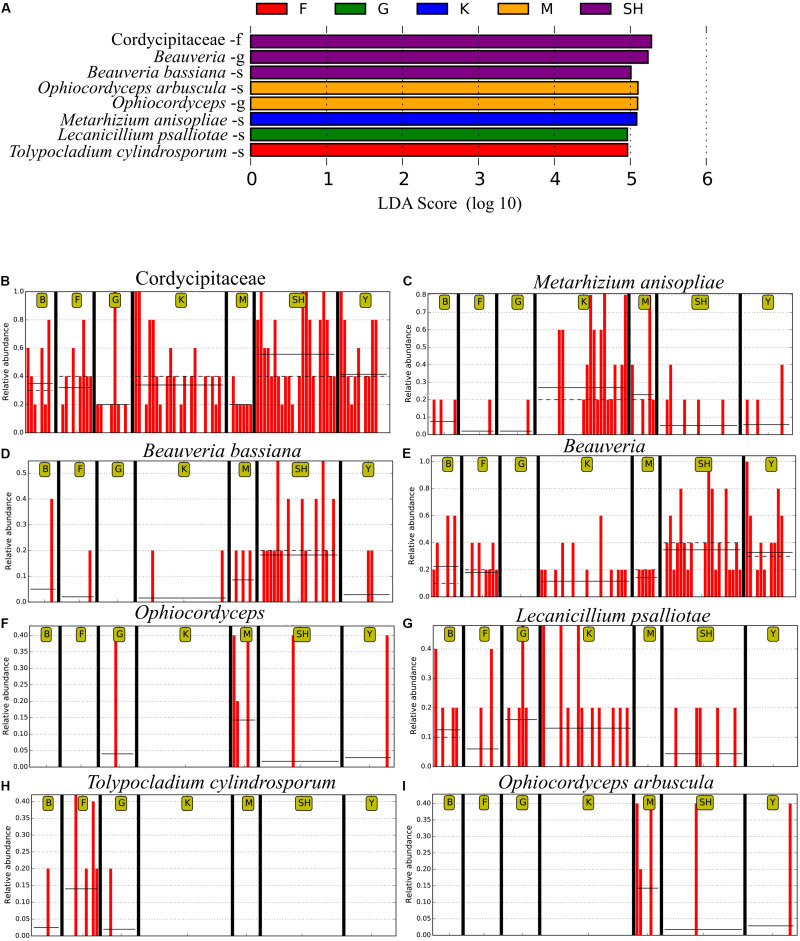
The LEfSe analysis of abundance detected eight operational taxonomic units (OTUs) that were over-represented among the sampling sites. **(A)** The taxonomic assignment of each OTU has been noted. The output of LEfSe analysis has been depicted by the linear discrimination analysis (LDA) score. Horizontal bars show the main taxonomic units with a significant LDA cutoff value ≥ 4 with *P* < 0.05, which were considerably altered among the sampling locations using LEfSe analysis. Red vertical bars show the relative abundance of **(B)** *Cordycipitaceae*, **(C)** *Metarhizium anisopliae*, **(D)** *Beauveria bassiana*, **(E)** *Beauveria*, **(F)** *Ophiocordyceps*, **(G)** *Lecanicillium psalliotae*, **(H)** *Tolypocladium cylindrosporum*, and **(I)** *Ophiocordyceps arbuscula*. SH (Taihang Mountains), K (Meishan to Ping Ke Xian), Y (Ya’an), M (Meishan), F (Saihanba National Forest Park; forest), G (Saihanba National Forest Park; grassland), and B (Saihanba National Forest Park; the boundary of forest and grassland). –f, family; -g, genus; -s, species.

In order to determine whether the community compositions were statistically different, we conducted an ANOSIM. The results indicated that the structure of insect–fungal pathogen communities at the K collection site differed from that of the collection sites at F (*R* = 0.1752; *P* = 0.013), SH (*R* = 0.1960; *P* = 0.001), Y (*R* = 0.1695; *P* = 0.003), and G (*R* = 0.2112; *P* = 0.004). There were differences between the structure of the fungal pathogen communities at the M collection site and those of the collection site at B (*R* = 0.3783; *P* = 0.006), F (*R* = 0.4846; *P* = 0.002), G (*R* = 0.5528; *P* = 0.003), and Y (*R* = 0.3139; *P* = 0.004). Besides the differences mentioned above, marked differences were observed between the G collection site and the Y collection site (*R* = 0.1701, *P* = 0.013) and SH (*R* = 0.1934, *P* = 0.01) (Supplementary Table S5). In addition, distance boxplots of the weighted- and the unweighted- UniFrac matrices were used to evaluate the diversity between sampling groups. The unweighted UniFrac results indicated that the K sampling site showed the highest variation, while the weighted UniFrac results indicated that the G collection showed the maximum variation (Figures 5A,B). The NMDS plot showed that the sample distribution of OTUs at the various collection sites was relatively concentric (Figure 5C). The weighted UniFrac and the unweighted UniFrac phylogenetic beta diversity yielded substantially different results regarding the structure of insect–fungal pathogen diversity. The results of the weighted UniFrac analysis suggested two main clusters, with the SH collection site forming a separate cluster. By contrast, the results of the unweighted UniFrac analysis suggested three main clusters (Figures 5D,E). Grouping the B and the F collection sites together using both the weighted- and the unweighted- phylogenetic beta diversity revealed a relative similarity between the insect–fungal pathogen communities at both collection sites. We compared the collection sites at the family level using *t*-test and found that the SH collection site differed from the collection sites F (*P* = 0.040), G (*P* = 0.006), K (*P* = 0.019), and M (*P* < 0.001) for the *Cordycipitaceae* family. Furthermore, the SH collection site differed from the F collection site (*P* = 0.026) for the *Ophiocordycipitaceae* family (Supplementary Figure S3). At the genus level, the relative frequencies of *Beauveria*, *Lecanicillium*, *Isaria*, and *Tolypocladium* differed at eight, five, two, and one pairwise comparison, respectively. More specifically, the results of the pairwise comparisons showed that the frequency of *Beauveria* was distinct for the pairs of F *vs*. G (*P* = 0.01), K *vs*. G (*P* = 0.001), K *vs*. Y (*P* = 0.036), SH *vs*. M (*P* = 0.007), M *vs*. G (*P* = 0.008), SH *vs*. G (*P* ≤ 0.001), Y *vs*. G (*P* = 0.002), and SH *vs*. K (*P* = 0.002). The abundance of *Lecanicillium* was statistically different for the pairs of K *vs*. M (*P* = 0.001), K *vs*. Y (*P* = 0.012), SH *vs*. M (*P* = 0.025), M *vs*. B (*P* = 0.049), and SH *vs*. K (*P* = 0.046). The frequency of *Isaria* genus was different for the pairs of SH *vs*. B (*P* = 0.015) and SH *vs*. K (*P* = 0.033). The abundance of *Tolypocladium* was significantly different between the SH and the K sampling sites (*P* = 0.040) (Supplementary Figure S4). At the OTU level, *Lecanicillium psalliotae*, *M. anisopliae*, *B. bassiana*, *Lecanicillium fungicola*, and *Metarhizium marquandii* differed at six, five, four, four, and three pairwise comparisons, respectively (Supplementary Figure S5). LDA, coupled with effect size measurement (LEfSe), was used in order to detect differentially abundant EF taxa from different sampling sites that may serve as biomarkers (Figure 6). The results showed that, at the family level, *Cordycipitaceae* (*P* < 0.05) was the discriminator in the soil samples of the SH collection site. Moreover, at the genus level and at the species level, *Beauveria* (*P* < 0.05) and *B. bassiana* (*P* < 0.01) were over-represented at the SH collection site, respectively. When all sites were investigated, *Ophiocordyceps* and *Ophiocordyceps arbuscula* of the M collection site emerged as discriminators at the genus level and at the species level, respectively. At the species level, *L. psalliotae* (*P* < 0.05) and *Tolypocladium cylindrosporum* (*P* < 0.01) were over-represented in the soil samples of the G and the F collection sites, respectively (Figures 6A,B).

### The Influence of Geographical Coordinates on the Composition of Fungal Pathogenic Communities

**FIGURE 7:**
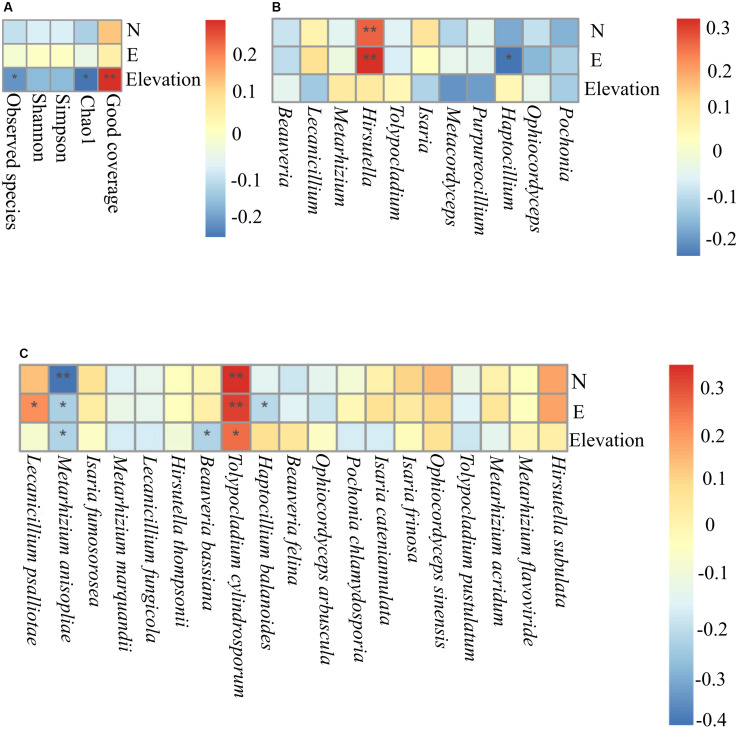
The Spearman association analysis of the relative frequency of the Alpha diversity indices **(A)**, the relative abundance of the dominant entomopathogenic fungi genera **(B)**, and species **(C)** with geographical coordinates. N, latitude; E, longitude **, adjusted *P* < 0.01; *, adjusted *P* < 0.05.

Trends among alpha diversity indices, dominant genera, and species were analyzed using Spearman’s rank correlation coefficients. This analysis demonstrated that elevation was a significant factor that positively correlated with good coverage (*P* = 0.0078). Elevation was negatively correlated with Chao1 (*P* = 0.012) and observed species (*P* = 0.034) (Figure 7A). At the genus level, *Haptocillium* (*P* = 0.026) was negatively correlated with longitude (102°35′.042′′ E). *Hirsutella* tended to show a significant positive correlation with longitude (E) (*P* = 0.00082) and latitude (42°32′.347′′ N) (*P* = 0.0057; Figure 7B). At the species level, *M. anisopliae senso lato* (*s.l.*, in the broad sense; 42°32′.347′′ N, *P* = 0.00001; 29°55′.457′′ E, *P* = 0.0139; 1,488 m elevation, *P* = 0.0161), *B. bassiana* (2,153 m elevation, *P* = 0.0153), and *Haptocillium balanoides* (109°32′.665′′ E, *P* = 0.026) were negatively correlated with certain geographical coordinates at different levels of significance. It was found that the relative frequency of *Tolypocladium cylindrosporum* was associated with latitude (42°33′.973′′ N, *P* = 0.00055), longitude (117°16′.647′′ E, *P* = 0.016), and elevation (1,561 m, *P* = 0.0119), while the relative abundance of *L. psalliotae* was positively correlated with longitude (29°55′.457′′ E, *P* = 0.039) (Figure 7C). The Beta diversity (Bray–Curtis distances) using a dbRDA displayed no significant relationship between the family level and the GPS coordinates (dbRDA envfit, N *R*^2^ = 0.0013, *P* = 0.9340; E *R*^2^ = 0.0064, *P* = 0.7156; elevation *R*^2^ = 0.0138, *P* = 0.5062). This analysis demonstrated the significant effect of latitudinal (dbRDA envfit, *R*^2^ = 0.1771, *P* = 0.001) and longitudinal ranges (dbRDA envfit, *R*^2^ = 0.1871, *P* = 0.0005) at the genus level. The effect of the elevational range was not significantly correlated (dbRDA envfit, *R*^2^ = 0.0094, *P* = 0.6321). The effect of the three factors, latitude (dbRDA envfit, *R*^2^ = 0.3004, *P* = 0.0005), longitude (dbRDA envfit, *R*^2^ = 0.1885, *P* = 0.0005), and elevation (dbRDA envfit, *R*^2^ = 0.2865, *P* = 0.0005), was significant at the species level (Supplementary Figure S6). Community dissimilarity increased significantly with geographical distance (the Mantel correlation = 0.228, *P* = 0.001 on 999 permutations).

### Natural Distribution and Phylogenetic Diversity Using the Baiting Method

**FIGURE 8:**
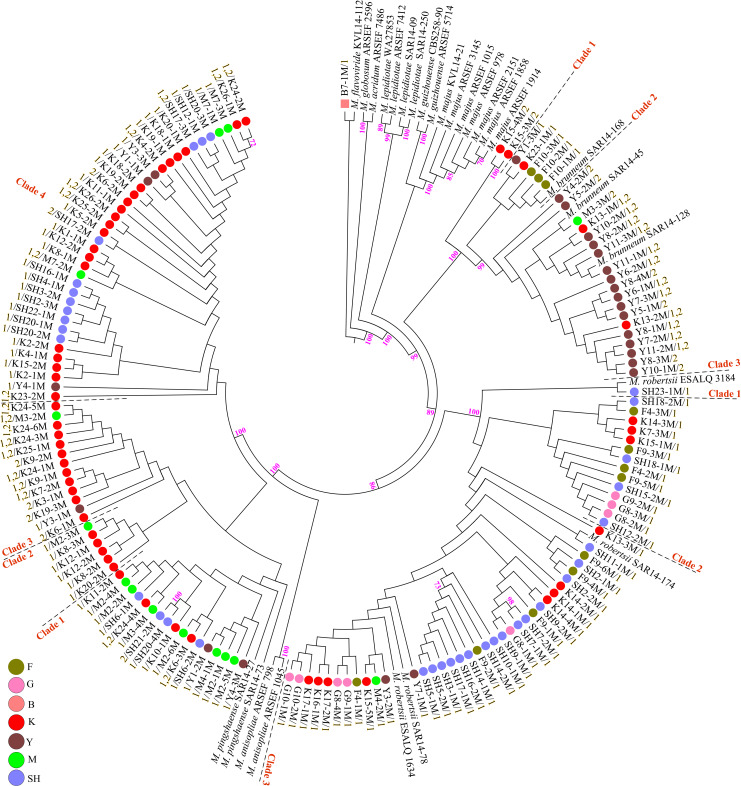
Maximum likelihood phylogeny of the *Metarhizium* isolates from the analysis of Mz_FG543 sequences. Support values were obtained from 1,000 bootstrap replicates, and bootstrap values have been denoted above the branches receiving ≥ 70% support. Branch terminals list the isolate name and mating type (MAT1-1-1 = 1/MAT1-2-1 = 2). Reference sequences were taken from the studies of Kepler et al. (2013, 2014), Keyser et al. (2015), and Castro et al. (2016). In the phylogenetic tree, the scale bar characterizes the number of anticipated alterations per site. ARSEF (USDA-ARS Collection of Entomopathogenic Fungal Cultures, Ithaca, NY, United States), CBS (Centraalbureau voor schimmelcultures Fungal Biodiversity Center, Utrecht, Netherlands), SAR (laboratory collection of Dr. Stephen A. Rehner, Beltsville, MD, United States), ESALQ (Escola Superior de Agricultura “Luiz de Queiroz” at University of São Paulo), KVL (Chad A. Keyser and Nicolai V. Meyling, University of Copenhagen, Copenhagen, Denmark), and WA (University of Warsaw, Warsaw, Poland). SH (Taihang Mountains), K (Meishan to Ping Ke Xian), Y (Ya’an), M (Meishan), F (Saihanba National Forest Park; forest), G (Saihanba National Forest Park; grassland), and B (Saihanba National Forest Park; the boundary of forest and grassland).

**FIGURE 9:**
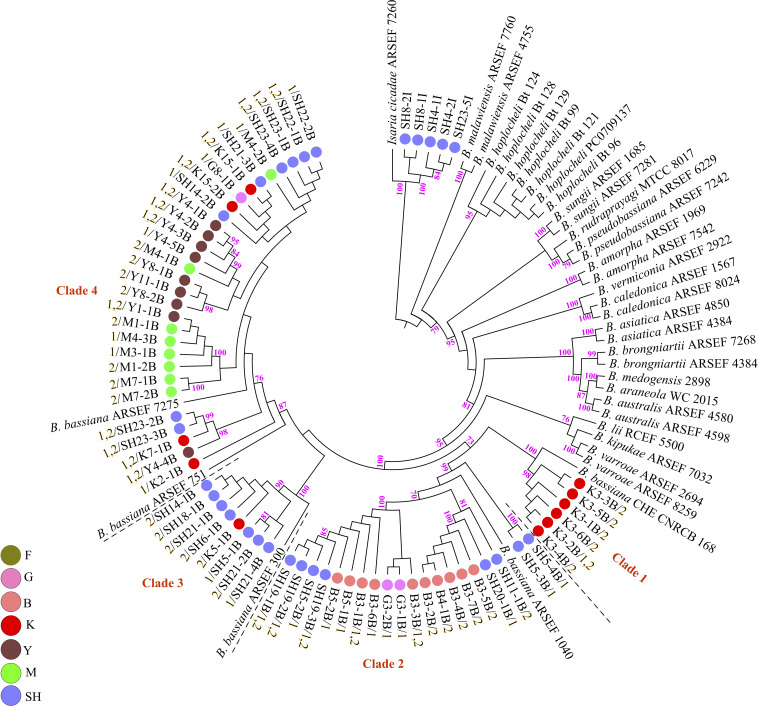
Maximum likelihood analysis of Biogenesis of Lysosomal Organelle Complex sequence data. Nodes that received ≥ 70% bootstrap support values are shown. In the phylogenetic tree, the scale bar signifies the number of predictable alterations per site. The tree has been rooted with *Isaria cicadae* (*Cordyceps cicadae*). Branch terminals list the isolate name and the mating type (MAT1-1-1 = 1/MAT1-2-1 = 2). Reference sequences were obtained from Rehner et al. (2011); Zhang et al. (2013), Agrawal et al. (2014); Robène-Soustrade et al. (2015), Imoulan et al. (2016); Chen et al. (2017), and Serna-Domínguez et al. (2018). ARSEF (USDA-ARS Collection of Entomopathogenic Fungal Cultures, Ithaca, NY, United States), CHE-CNRCB (Colección de hongos entomopatógenos del Centro Nacional de Referencia de Control Biológico, Mexico), and RCEF (Research Center on Entomogenous Fungi, Anhui Agricultural University, Hefei, China). SH (Taihang Mountains), K (Meishan to Ping Ke Xian), Y (Ya’an), M (Meishan), F (Saihanba National Forest Park; forest), G (Saihanba National Forest Park; grassland), and B (Saihanba National Forest Park; the boundary of forest and grassland).

Fungal isolation based on standard soil baiting with the *T. molitor* larvae resulted in the isolation of 161 *Metarhizium*, 64 *Beauveria*, and five *Isaria* isolates from the soil samples collected at the 98 forest sites and grassland sites (Table 2). The overall distribution of the *Metarhizium* and *Beauveria* isolates by area was found to be significantly heterogeneous (χ^2^ = 39.217; df = 4; *P* < 0.0001) and homogenous (χ^2^ = 11.313; df = 4; *P* = 0.0233), respectively. All fungal isolates were sub-cultured and phylogenetically assigned to the species level using Mz_FG543 and Bloc loci for *Metarhizium* and *Beauveria*, respectively (Figures 8, 9). The Bloc region for the five *Isaria* isolates was amplified successfully and aligned with the *Beauveria* reference sequences for phylogenetic assignment (Figure 9). A total of four species lineages (*Metarhizium pingshaense*, *Metarhizium robertsii*, *Metarhizium brunneum*, and *M. flavoviride*) were identified in *Metarhizium*, and their phylogenetic placement has been shown (Figure 8). Furthermore, nine *Metarhizium* isolates were obtained from the SH location (SH3 sampling site) (Table 2). The initial morphological characterizations revealed the identity of these nine isolates as belonging to the *M. flavoviride* species complex, where Mz_FG543 primers failed to amplify the intergenic spacer region of those isolates. Amplification and sequencing of the 5’ elongation factor-1 alpha region (5′-TEF) were accomplished for these isolates according to the procedure of Kepler et al. (2014). The result suggested a potential new sister cluster with the *Metarhizium pemphigi* clade within the *M. flavoviride* species complex (Supplementary Figure S7). The sequence types of the 5’-TEF gene fragment were submitted to GenBank (Supplementary Table S2). The maximum likelihood of the phylogeny of Mz_FG543 was topologically congruent and consistent with that of prior analyses (Bischoff et al., 2009; Kepler and Rehner, 2013; Kepler et al., 2014; Rehner and Kepler, 2017). The backbone and the monophyly of the PARBH species complex (*M. pingshaense*, *M. anisopliae, M. robertsii*, *M. brunneum*, and *M. humberi*) were strongly supported by the phylogenetic assessment of the Mz_FG543 locus with *M. robertsii*, the sister to a clade containing *M. anisopliae*, *M. pingshaense*, and *M. brunneum*, creating the basal-most clade (Rehner and Kepler, 2017; Luz et al., 2019). *M. pingshaense* was the most common species, with 74 isolates. It was divided into four major subclades and subsequently referred to as the *M. pingshaense* subclades 1 (*n* = 17), 2 (*n* = 6), 3 (*n* = 13), and 4 (*n* = 38) (Figure 8). Sublades 2, 3, and 4 were not accompanied by any *M. pingshaense* reference sequences. The second most abundant species was found to be *M. robertsii*, with 51 isolates, and it was subgrouped into three major subclades, subsequently referred to as *M. robertsii* subclades 1 (*n* = 1), 2 (*n* = 14), and 3 (*n* = 36) (Figure 8). Subclade 2 was not clustered with the *M. robertsii* reference sequences. A majority of the terminal and internal branches of both the *M. robertsii* and the *M. pingshaense* isolates were unsupported and formed shallow grades. The two remaining species, *M. brunneum* and *M. flavoviride*, were composed of 26 isolates and one isolate, respectively. The *Metarhizium brunneum* clade was divided into three intraspecific phylogenetic subclades, subsequently referred to as *M. brunneum* subclade 1 (*n* = 2), 2 (*n* = 5), and 3 (*n* = 19). Subclades 1 and 3 were not grouped with any *M. brunneum* reference sequences. The phylogenetic diversity and the relationships among *Isaria* and *Beauveria* isolates were evaluated *via* the molecular phylogeny of the Bloc locus. All *Beauveria* isolates were placed within the *B. bassiana* clade (Rehner et al., 2011) and further subdivided into four terminal subclades with a strong bootstrap support (Figure 9), whereas the majority of the terminal branches were unsupported. All four subclades were grouped with the *B. bassiana* reference sequences.

**TABLE 2:** Total number of insect–fungal pathogens obtained from the soil samples collected at 98 locations in China.

Fungal species	Locations						
	SNFP						
	F	B	G	SH	K	M	Y
*M. pingshaense*	0	0	0	15	41	12	6
*M. robertsii*	9	0	8	20	11	1	2
*M. brunneum*	3	0	0	0	5	1	17
*M. flavoviride*	0	1	0	0	0	0	0
*Metarhizium* spp.	0	0	0	9	0	0	0
*B. bassiana*	0	10	3	23	11	8	9
*Isaria* spp.	0	0	0	5	0	0	0

*SNFP, Saihanba National Forest Park; F, forest region; B, the boundary of forest and grassland area; G, grassland; SH, Taihang Mountains; K, Meishan to Ping Ke Xian; M, Meishan; Y, Ya’an region. The numbers represent the fungal isolates obtained by insect baiting using *Tenebrio molitor* larvae.*

### Determination of Mating Type (Mating Type Assignment)

PCR mating-type assignments revealed the presence of either MAT1-1-1 or MAT1-2-1 in both the *Metarhizium* isolates and the *Beauveria* isolates (Supplementary Figures S8, S9). All the *M. robertsii* isolates were found to possess only the MAT1-1-1 idiomorph. Some isolates of *M. brunneum* and *M. pingshaense* demonstrated both mating types in a single isolate. The single isolate of *M. flavoviride* was found to possess a single mating type (MAT1-1-1). Of the 26 *M. brunneum* isolates, five isolates were found to possess just MAT1-1-1 (*M. brunneum*, subclade 2), nine isolates were found to possess just MAT1-2-1 (*M. brunneum*, subclades 1 and 3), and 12 isolates demonstrated both mating types (*M. brunneum*, subclade 3). Notably, the three *M. brunnuem* isolates from SNFP were found to possess MAT1-1-1 (*M. brunneum* Subclade 2) (Figure 8). In contrast, isolates of *M. brunneum* from Sichuan Province showed either MAT1-1-1, MAT1-2-1, or both. Of the 74 *M. pingshaense* isolates, 49 were found to possess MAT1-1-1, seven were found to possess MAT1-2-1, and 18 isolates showed both mating types. The MAT1-1-1 idiomorph was distributed in the four *M. pingshaense* subclades (Figure 8). The *Beauveria* isolates containing only the MAT1-2-1 idiomorph were distributed in subclade 3, while those containing both mating types were distributed in subclades 1, 3, and 4. Of the 64 *B. bassiana* isolates, 20 were found to possess the MAT1-1-1 idiomorph (subclades 2, 3, and 4), 25 were found to possess only the MAT1-2-1 idiomorph (subclades 1–4), while 19 showed both mating types (subclades 1, 2, and 4) (Figure 9).

## Discussion

To the best of our knowledge, the current study utilized both insect baiting and HTS techniques to provide the most comprehensive analysis of the natural distribution of insect-associated fungi in forest habitats and grassland habitats to date. Our results demonstrated that *Metarhizium* was the most dominant genus, occurring most frequently at all collection sites except at the SH location. *Beauveria* was the most predominant genus at the SH collection site. Species abundance distribution (SAD) is one of the most basic models used in studies associated with population ecology. SAD describes communities that contain a few highly numerous species and many uncommon species. It is considered as a general pattern in ecology (Shade et al., 2018), and insect-associated fungi are not excluded from this comprehensive regulation. Similarly, our HTS results indicated that the community structure of EFs was composed of some highly abundant genera (*Metarhizium*, *Beauveria*, and *Isaria*) and several rare taxa such as *Lecanicillium*, *Tolypocladium*, *Purpureocillium*, *Hirsutella, Haptocillium*, and *Ophiocordyceps* (Figures 1C,D). The three leading hypocrealean families are composed of genera and species which regularly colonize multiple hosts, varying from plants, nematodes, and fungi to a broad range of insects (Kepler et al., 2012, 2014; Araújo and Hughes, 2016). In addition to the commonly observed species *M. anisopliae s.l.* and *B. bassiana*, this work detected other invertebrate pathogens within these three families *via* deep sequencing of the soil samples. For instance, the *Pochonia* genus comprised of species that not only show pathogenicity to nematodes but also infect mollusk eggs and rotifers (Zare and Gams, 2001) and demonstrate a capability to colonize the soil rhizosphere of plants (Maciá-Vicente et al., 2009; Moonjely and Bidochka, 2019). In the current study, *P. chlamydosporia* was detected with a frequency of 1.7 and 3% at the SH and the K sampling collection sites, respectively (Figure 4). Other invertebrate pathogens, such as *M. marquandii* (*Paecilomyces marquandii*), which was mostly predominant (6.1%) at the K collection site, were also detected (Figure 4). The nematicidal activity of *M. marquandii* has been reported in a previous study (Genier et al., 2016). However, this taxon is not a member of the core *Metarhizium* clade generally considered for biocontrol programs (Kepler et al., 2014). The pathogens of nematodes also exist in other taxa, which are classified outside of *Clavicipitaceae*, such as some species of *Hirsutella* and *Haptocillium* (Kepler et al., 2014; Simmons et al., 2015). *Hirsutella* (asexual morphs) species are classified as a host-specific entomopathogenic soil fungus. These species are physiologically and morphologically distinct from *Metarhizium* and *Beauveria*, which have over 700 hosts (Qu et al., 2018). *Hirsutella thompsonii* is a widely studied species from this genus, which is potentially pathogenic toward mites (Tigano et al., 2006). The determination of OTUs at the species level detected two species of this genus: *H. thompsonii* and *Hirsutella subulata*. The former was detected at the SH (1.7%) and at the M (8.5%) collection sites, whereas the latter was detected at the SH (0.8%), the F (2%), and the G (2%) collection sites (Figure 4). The genus *Lecanicillium* contains both entomogenous and fungicolous species (Berendsen et al., 2010) and is commonly found in the tropical and the temperate regions (Pearce et al., 2009). The *Lecanicillium fungicola* and *L. psalliotae* species from this genus were detected with HTS technique and were present in three sampling sites and five sampling sites, respectively, while also differing in prevalence at their respective sites (*L. fungicola* 45.99% *vs*. *L. psalliotae* 51.92%) (Figure 4). It has been suggested that *L. fungicola* shows potential for pathogenicity against insects (Berendsen et al., 2010). This may be due to the ability of this fungus to infect mushrooms and the close relatedness of this species to a variety of insect pathogens (Amey et al., 2007; Berendsen et al., 2010). Interestingly, *L. psalliotae*, which was initially recognized as a pathogen of mushrooms (Treschow, 1941), is presently most often discussed in relation to its nematocidal activity (Pirali-Kheirabadi et al., 2007). We should mention that the species-level assignment (Figure 4) must be interpreted with some caution because using ITS2 for soil–fungal entomopathogen composition structures does not provide informative molecular delimitation below the genus level accurately, especially for the closely related species in some genera such as *Metarhizium* and *Beauveria*, and that is a straightforward consequence of the dependence of culture-independent metabarcoding approach on databases. For this reason, proper identification should be integrated with additional standard techniques (Tremblay et al., 2019).

In this study, *Metarhizium* and *Beuaveria* were relatively abundant across all sampling sites. Prior studies that have laid emphasis on the ability of these generalist fungi to interact with plants, suggesting that the type of vegetation canopy may be associated with their widespread distribution in ecosystems (Wyrebek et al., 2011; Behie et al., 2012; Behie and Bidochka, 2013; Kepler et al., 2015; Keyser et al., 2015; McKinnon et al., 2017, 2018; Tall and Meyling, 2018). Thus, based on the unique relationship of *Metarhizium* and *Beauveria* lineages with the plant rhizospheres, the high frequencies with which these are found in the forest habitats and the grassland habitats can be conceivably illustrated by adding an analysis of fungal–plant associations to the study. Bidochka et al. (2005) hypothesized that the global distribution and the abundance models of *Metarhizium* lineages may be based on two central principles. In temperate regions, fungal genotypes may be linked with habitats characterized by specific abiotic environmental determinants that select for the genotypes that may be present (habitat selection), while in the tropical and in the subtropical regions, fungal genotypes may be associated with particular hosts (host selection). Bidochka et al. (2002) expanded the habitat selection hypothesis for entomopathogenic soil fungi by including the association between *B. bassiana* distribution and habitat availability. They contended that the congruence between *B. bassiana* lineages and habitat nature was rationally indistinguishable from that of the *M. anisopliae* lineages (Bidochka et al., 2001), in which two distinct genetic groups (*M. robertsii* and *M. brunneum*) were found to be associated with different localities. Such circumstantial evidence presented by these studies represent a significant paradigm shift, wherein it is habitat selection, not insect host selection, that determines the community composition of insect–pathogenic fungi (Bidochka et al., 2001, 2002). The four *Metarhizium* species obtained using the baiting method in this study were not consistently distributed across the investigated collection sites. *M. pingshaense* was the most common species obtained from the soil collection sites (*n* = 74), followed by *M. robertsii* (*n* = 51), *M. brunneum* (*n* = 26), and *M. flavoviride* (*n* = 1). *M. pingshaense* was obtained throughout the investigated sites except at the SNFP collection site and was isolated from 45 samples of the total 490 soil samples. By contrast, *M. brunneum*, which was recovered from 15 soil samples and since it was not isolated from the G, the B, and the SH collection sites, was more narrowly distributed. These results are in line with several investigations from agricultural soils that have confirmed the locality-specific abundance and distribution of the *Metarhizium* lineages, at least in northern Europe (i.e., Denmark) (Meyling et al., 2011; Steinwender et al., 2014, 2015; Keyser et al., 2015). The *M. robertsii* isolates were obtained from all seven collection sites except the boundary between the grassland and the forest regions (B). Surprisingly, our baiting results, based on the infection of mealworm larvae, highlighted that the *M. pingshaense* lineages had a higher abundance and frequency compared to those of the *M. robertsii* lineages, although the *M. robertsii* lineages had a better distribution and a wider occurrence across the sampling sites (Table 2). The HTS results verified the highest frequency of the *Metarhizium* lineages from the G (grassland) collection site (56.0%) (Figure 1A), and the intergenic marker phylogeny indicated that all the *Metarhzium* isolates obtained from this site belonged to the *M. robertsii* clade (Figure 8). These results might lead us to conclude that the distribution of the *M. pingshaense* lineages was restricted to forest regions with a higher average annual temperature (Supplementary Table S1), while the *M. robertsii* lineages can stand the relatively extreme environmental temperatures and may possibly possess better ecological fitness in different environments. In addition to the abiotic theory, *M. robertsii* could be more closely associated with grassland habitats (Wyrebek et al., 2011). The average annual temperature at SNFP is approximately −1.3°C, with a prolonged cold winter and a brief growing season (May to September) (Yang et al., 2014). The capability of *Metarhizium* for cold tolerance was assessed, and Fang and St. Leger (2010) reported that the *Metarhizium* lineages acquired the cold shock response genes (CRP1 and CRP2) evolutionarily *via* horizontal gene transfer from soil-dwelling bacteria within the same ecological niche. The low level of the *Metarhizium* isolation at the SNFP sampling site (Table 2) encouraged us to perform a selective agar medium to evaluate the *Metarhizium* frequency according to the procedure of Keyser et al. (2015). The results (not shown here) showed that the number of colony-forming units per plate (CFU/plate) was higher at the F sampling sites, whereas at the B and the G sampling sites the number of CFU was lower. These results were not consistent with the HTS and the baiting results, suggesting that different isolation methods resulted in various fungal frequencies. As highlighted by other studies, using a single detection method raises many doubts regarding the abundance of fungal pathogens in the environment (Keller et al., 2003; Goble et al., 2010; Medo and Cagáň, 2011). During the course of the study, we considered these procedural deficiencies, and the baiting approach may not be genuinely quantitative (Jaronski, 2007; Scheepmaker and Butt, 2010). Due to this reason, we performed HTS to provide an additional dimension that facilitated a deeper understanding of the insect-associated fungal distribution. In contrast to the predominance of the *M. anisopliae* species complex observed in the sampling sites, we reported a potentially new sister clade to the *M. pemphigi* cluster composed of nine fungal isolates within the *M. flavoviride* species complex using the sequence of the 5′-TEF gene fragment (Supplementary Figure S7). These isolates were only obtained from the SH collection site, indicating locality-specific abundance and distribution of these isolates, and further studies are needed to understand the potential interactions of this clade with hosts (plant roots and insects) and its above- and below-ground ecology. Our study indicated that both *M. brunneum* and *B. bassiana* were likely to be obtained from the site with a relatively high elevation (2,447 m) and from regions with elevations below (300–100 m). Our major finding for *M. brunneum* in terms of elevation is substantiated by those of Masoudi et al. (2018) in China but notably at odds with those of Inglis et al. (2019) in Canada. The highest elevation from which *M. pingshaense* and *M. robertsii* were obtained by the current study was 1,120 and 1,631 m, respectively. Inglis et al. (2019) isolated *M. brunneum* at a latitude of 55° N and the highest elevation of 1,012 m. Masoudi et al. (2018) obtained *M. pingshaense* from the Qinling Mountains (33° N) at an elevation of 3,000 m. Previous studies have reported the isolation of the *Metarhizium* lineages from latitudes as high as 67° N in Finland (Vänninen, 1996) and 64° N in Iceland (Oddsdóttir et al., 2010). Our HTS data indicated a negative correlation between *M. anisopliae s.l.* lineages and geographical coordinates (42°32′.347′′ N, 29°55′.457′′ E) (Figure 7). A few studies have reported that latitude was correlated with *B. bassiana* distribution, while longitude was correlated with *M. anisopliae* natural distribution (Bidochka et al., 1998; Quesada-Moraga et al., 2007; Garrido-Jurado et al., 2011). Our HTS results and, the Mantel tests verified that community dissimilarities increased significantly with geographical distance (the Mantel correlation = 0.228, *P* = 0.001). Thus, we inferred that geographical coordinates may be the factors that possibly influence insect–fungal pathogen community composition in the investigated sites. Data gathered by natural occurrence studies using bait insect species possibly underestimate not only the diversity and frequency of commonly occurring EF species but also that of rare taxa, such as the hypocrealean insect pathogens (Vänninen, 1996; Chandler et al., 1997; Bidochka et al., 1998, 2001; Klingen et al., 2002; Ali-Shtayeh et al., 2003; Keller et al., 2003; Meyling and Eilenberg, 2006; Quesada-Moraga et al., 2007; Inglis et al., 2008, 2019; Sun et al., 2008; Goble et al., 2010; Medo and Cagáň, 2011; Meyling et al., 2011; Rocha et al., 2013; Wakil et al., 2013; Steinwender et al., 2014; Kepler et al., 2015; Keyser et al., 2015; Rezende et al., 2015; Hernández-Domínguez and Guzmán-Franco, 2017). Some of these studies have reported lineages of *Metarhizium*, *Beauveria*, and *Isaria* as the common pathogenic microorganisms found in soil environments (Table 1). The results of our HTS proved that *M. anisopliae s.l.*, *B. bassiana*, and *I. farinosa* were the core insect-associated fungal genera in the assessed habitats (Figures 3, 4). The results of HTS help us to distinguish the difference between ‘locally abundant’ OTUs and the ‘core’ (frequently abundant) diversity of fungal pathogens in the soil. Moreover, the results of the baiting method showed that the *Isaria* isolates were only obtained from the SH collection sites (SH4, SH8, and SH23) (Figure 9). Furthermore, Krona analysis indicated that the frequencies of *Isaria* at SH4 (two isolates), SH8 (two isolates), and SH23 (one isolate) sampling sites were 88, 65, and 73%, respectively (Supplementary Figures S10–S12). Also, *B. bassiana* (four isolates) was obtained from the SH23 sampling site with a frequency of 17% (Supplementary Figure S11). These results indicate that the detection of pathogenic microorganisms using the baiting method may be strongly influenced by two factors, which are fungal frequency and the potential entomopathogenicity of the pathogens. The co-occurrence of *B. bassiana* and the *Isaria* sp. was detected at the SH23 sampling site, where the relative frequency of *B. bassiana* was less than that of the *Isaria* sp., but the number of infected cadavers with *Beauveria* was four times higher than that infected with *Isaria*. This result showed that, compared to the *Isaria* lineages, the *B. bassiana* isolates demonstrated superior virulence even when present at a lower frequency (17%) and exhibited this virulence by competing for hosts and by infection of more insects. These two factors may confer a better competitive capability on the pathogen for colonizing insect larvae, which enables it to exclude and/or affect the presence of other pathogens. Based on these data, it may be inferred that the negative interaction between pathogens caused by the co-occurrence of other fungal pathogens in soils may lead to direct competition in which the subdominant taxa may be excluded from the outcome of baiting (competitive exclusion) (Gold et al., 2009). Additionally, it has been suggested that each bait insect species method may highlight the discriminating isolation of specific fungal species/taxa (Klingen et al., 2002; Goble et al., 2010) under a particular environmental condition (Mietkiewski and Tkaczuk, 1998). Klingen et al. (2002) highlighted that the fungal species obtained varied significantly depending on the insect species used as bait. For example, their results showed that *T. cylindrosporum* and *B. bassiana* were obtained only using *Delia floralis* (Diptera: Anthomyiidae) and *G. mellonella*, respectively (Klingen et al., 2002) (Table 1). Our HTS data detected that *T. cylindrosporum* followed the same frequency trend of the SNFP sampling location, F > B > G (14 > 2.5 > 2% for all the three families of the EF OTUs, respectively) and detected *T. pustulatum* only at the K (2.3%) and at the Y (2.8%) collection sites. These results indicate that some *Tolypocladium* species may possibly have a limited geographical distribution, although *Tolypocladium* sp. was detected at all locations except at the F sampling site (Figure 4). This may be a conceivable reason for the failure of our insect bait method, which was similar to the methods used by Steinwender et al. (2014); Keyser et al. (2015), and Korosi et al. (2019), to detect other hypocrealean fungi such as *Lecanicillium*, *Hirsutella*, and *Tolypocladium*. Environmental factors can possibly underlay EFs habitat preference. Jaronski (2007) provided a meticulous overview of the soil physicochemical ecology in relation to hypocrealean insect-associated fungi and concluded that the complex interactions of fungal pathogens with the abiotic parameters of soil may exert both negative as well as positive effects on the community structure of soil-inhabiting entomopathogens. In order to develop a comprehensive picture of insect-associated fungal diversity in soils, additional studies that investigate the influence of edaphic parameters (physicochemical properties) on the abundance and the occurrence of EFs in soil environments may be needed.

The current study explored and compared the hypocrealean EF isolates (*Metarhizium* and *Beauveria*) that were obtained using mating-type PCR screening that addressed their potential for sexual reproduction and verified their distribution on the phylogenetic tree (Figures 8, 9). Mating-type PCR assays revealed that some isolates of *M. pingshaense*, *M. brunneum*, and *B. bassiana* contained both mating-type genes (homothallic). In a cutting edge global-scale survey, Rehner and Kepler (2017) reported the presence of a MAT1-1-1 or a MAT1-2-1 mating-type idiomorph in the *M. anisopliae* PARBH species complex, where they speculated that all PARBH species were fundamentally outcrossing (Rehner and Kepler, 2017). Results from the mid-Atlantic region showed that individual *M. robertsii* and *M. brunneum* strains possessed a single mating type, either MAT1-1-1 or MAT1-2-1, indicating that these species were heterothallic (Kepler et al., 2015). However, Pattemore et al. (2014) highlighted the existence of both MAT1-1-1 and MAT1-2-1 idiomorphs in the genome sequence of an Australian isolate of *M. anisopliae* (BRIP 53293, APNB00000000) and determined that the isolate was homothallic and thus potentially self-fertile. Rehner and Kepler (2017) reported that all the Australian *M. anisopliae* isolates in their study were of a single mating type, either MAT1-1-1 or MAT1-2-1. Mating-type characterization of our *B. bassiana* isolates showed that the 19 isolates possessed both idiomorphs. Meyling et al. (2009) reported that most *B. bassiana* clades within an agroecosystem in Denmark were fixed at a specific mating type, indicating that they retained the potential at least for heterothallic sexual activity, and that one population (Eu-1) contained two mating types, suggesting a potential for sexual reproduction at that study site for this population. Ramos et al. (2017) reported a skewed distribution of the mating-type system of MAT1/MAT2 (146/4) of the *B. bassiana* populations from Cuba, indicating a limited potential for the recombination of complementary mating specificity (or mating types). Our results of mating-type characterization of the obtained EF isolates indicate a major development, showing the possible sexual reproduction processes within entomopathogen species of the *M. anisopliae* PARBH complex and the *B. bassiana* populations inhabiting Chinese soils. The inclusion of the mating-type assignment PCR assays as a part of natural distribution surveys of hypocrealean entomopathogenic fungi may provide useful information related to the spatial distribution of mating-compatible individuals in the environment, thereby enabling the identification of populations in which the EF lineages experience occasional sexual recombination events with profound consequences for their population dynamics and evolutionary trajectories.

## Conclusion

Despite the abundance of information regarding the natural distribution of widespread EFs, such as *Metarhizium*, *Beauveria*, and *Isaria* lineages in different habitats, obtained *via* baiting methodology under laboratory conditions, there is a lack of understanding with respect to the frequency profiles of other EFs in soils, irrespective of certain circumstances, such as incubation temperature or the type of insect used as a bait, that may affect the isolation of the entomopathogens. This current study showed that the forests and the grasslands of two geographical regions of China harbored a relatively high frequency of *Metarhizium* and *Beauveria* and highlighted the abundance of those fungal pathogen taxa whose frequency or presence has not been frequently reported by other studies using HTS method for soil samples as well as the discovery of a potentially new cluster within the *M. flavoviride* species complex. The study indicates that the prevalence of insect fungal pathogens in certain habitats should be a greater focus of future efforts to address the factors influencing the locality-specific abundance and distribution of such fungi. This may lead to a better understanding of the dynamics and the distribution of EFs in order to recommend insect-associated fungi as a biocontrol agent.

## Data Availability Statement

The datasets generated for this study can be found in the raw sequences produced in the current study can be reached through the National Center for Biotechnology (NCBI) under the BioProject ID PRJNA551928, accession SUB5873947.

## Author Contributions

AM and HW originated the research. AM, MW, XZ, CW, HW, ZQ, and WW obtained the soil samples. AM performed the data analysis, the laboratory experiments, and wrote the manuscript. JL offered assistance in designing the research and improving the quality of the manuscript. All the authors corrected the first draft of the manuscript.

## Conflict of Interest

The authors declare that the research was conducted in the absence of any commercial or financial relationships that could be construed as a potential conflict of interest.

## Footnotes

1 http://data.cma.cn/data/online/t/1

2 www.thermofisher.com

3 https://www.ncbi.nlm.nih.gov

4 http://cutadapt.readthedocs.io/en/stable/

5 https://unite.ut.ee/

6 http://qiime.org/tutorials/denoising_454_data.html

7 www.graphpad.com

8 http://www.phylo.org

9 https://www.adobe.com/products/illustrator.html

## References

[B1] Abarenkov K. Henrik Nilsson R. Larsson K.-H. Alexander I. J. Eberhardt U. Erland S. (2010). The UNITE database for molecular identification of fungi – recent updates and future perspectives. *New Phytol.* 186 281–285. 10.1111/j.1469-8137.2009.03160.x 20409185

[B2] Agrawal Y. Mual P. Shenoy B. (2014). Multi-gene genealogies reveal cryptic species *Beauveria rudraprayagi* sp nov from India. *Mycosphere* 5 719–736. 10.5943/mycosphere/5/6/3

[B3] Ali-Shtayeh M. S. Mara’i A.-B. B. Jamous R. M. (2003). Distribution, occurrence and characterization of entomopathogenic fungi in agricultural soil in the Palestinian area. *Mycopathologia* 156 235–244. 10.1023/a:1023339103522 12749589

[B4] Altschul S. F. Gish W. Miller W. Myers E. W. Lipman D. J. (1990). Basic local alignment search tool. *J. Mol. Biol.* 215 403–410. 2231712 10.1016/S0022-2836(05)80360-2

[B5] Amey R. C. Athey-Pollard A. Mills P. R. Foster G. D. Bailey A. (2007). Investigations into the taxonomy of the mushroom pathogen *Verticillium fungicola* and its relatives based on sequence analysis of nitrate reductase and ITS regions. *Microbiology* 76 757–768. 10.1134/s0026261707060161 18297878

[B6] Araújo J. P. M. Hughes D. P. (2016). “Chapter one - diversity of entomopathogenic fungi: which groups conquered the insect body?” in *Advances in Genetics*, eds Lovett B. St. Leger R. J. (Cambridge, MA: Academic Press), 1–39. 10.1016/bs.adgen.2016.01.001 27131321

[B7] Behie S. W. Bidochka M. J. (2013). Potential agricultural benefits through biotechnological manipulation of plant fungal associations. *Bioessays* 35 328–331. 10.1002/bies.201200147 23319143

[B8] Behie S. W. Zelisko P. M. Bidochka M. J. (2012). Endophytic insect-parasitic fungi translocate nitrogen directly from insects to plants. *Science* 336 1576–1577. 10.1126/science.1222289 22723421

[B9] Berendsen R. L. Baars J. J. P. Kalkhove S. I. C. Lugones L. G. Wösten H. A. B. Bakker P. A. H. M. (2010). *Lecanicillium fungicola*: causal agent of dry bubble disease in white-button mushroom. *Mol. Plant Pathol.* 11 585–595. 20695998 10.1111/j.1364-3703.2010.00627.x PMC6640384

[B10] Bidochka M. J. Kamp A. M. Lavender T. M. Dekoning J. De Croos J. N. A. (2001). Habitat association in two genetic groups of the insect-pathogenic fungus *Metarhizium anisopliae*: uncovering cryptic species? *Appl. Environ. Microbiol.* 67 1335–1342. 10.1128/aem.67.3.1335-1342.2001 11229929 PMC92732

[B11] Bidochka M. J. Kasperski J. E. Wild G. A. (1998). Occurrence of the entomopathogenic fungi *Metarhizium anisopliae* and *Beauveria bassiana* in soils from temperate and near-northern habitats. *Can. J. Bot.* 76 1198–1204. 10.1139/b98-115 11771505

[B12] Bidochka M. J. Menzies F. V. Kamp A. M. (2002). Genetic groups of the insect-pathogenic fungus *Beauveria bassiana* are associated with habitat and thermal growth preferences. *Arch. Microbiol.* 178 531–537. 10.1007/s00203-002-0490-7 12420176

[B13] Bidochka M. J. Small C.-L. N. Spironello M. (2005). Recombination within sympatric cryptic species of the insect pathogenic fungus *Metarhizium anisopliae*. *Environ. Microbiol.* 7 1361–1368. 10.1111/j.1462-5822.2005.00823.x 16104859

[B14] Bischoff J. F. Rehner S. A. Humber R. A. (2009). A multilocus phylogeny of the *Metarhizium anisopliae* lineage. *Mycologia* 101 512–530. 10.3852/07-202 19623931

[B15] Boomsma J. J. Jensen A. B. Meyling N. V. Eilenberg J. (2014). Evolutionary interaction networks of insect pathogenic fungi. *Annu. Rev. Entomol.* 59 467–485. 10.1146/annurev-ento-011613-162054 24160418

[B16] Caporaso J. G. Kuczynski J. Stombaugh J. Bittinger K. Bushman F. D. Costello E. K. (2010). QIIME allows analysis of high-throughput community sequencing data. *Nat. Methods* 7 335–336. 20383131 10.1038/nmeth.f.303 PMC3156573

[B17] Castro T. Mayerhofer J. Enkerli J. Eilenberg J. Meyling N. V. Moral R. D. A. (2016). Persistence of Brazilian isolates of the entomopathogenic fungi *Metarhizium anisopliae* and *M. robertsii* in strawberry crop soil after soil drench application. *Agric. Ecosyst. Environ.* 233 361–369. 10.1016/j.agee.2016.09.031

[B18] Chandler D. Hay D. Reid A. (1997). Sampling and occurrence of entomopathogenic fungi and nematodes in UK soils. *Appl. Soil Ecol.* 5 133–141. 10.1016/s0929-1393(96)00144-8

[B19] Chao A. (1984). Nonparametric estimation of the number of classes in a population. *Scand. J. Stat.* 265–270.

[B20] Chen W.-H. Han Y.-F. Liang Z.-Q. Jin D.-C. (2017). A new araneogenous fungus in the genus *Beauveria* from Guizhou, China. *Phytotaxa* 302 57–64.

[B21] Choi Y.-W. Hyde K. D. Ho W. H. (1999). Single spore isolation of fungi. *Fungal Divers.* 3 29–38.

[B22] Edgar R. C. (2004). MUSCLE: multiple sequence alignment with high accuracy and high throughput. *Nucleic Acids Res.* 32 1792–1797. 10.1093/nar/gkh340 15034147 PMC390337

[B23] Edgar R. C. (2013). UPARSE: highly accurate OTU sequences from microbial amplicon reads. *Nat. Methods* 10 996–998. 10.1038/nmeth.2604 23955772

[B24] Edgar R. C. Haas B. J. Clemente J. C. Quince C. Knight R. (2011). UCHIME improves sensitivity and speed of chimera detection. *Bioinformatics* 27 2194–2200. 10.1093/bioinformatics/btr381 21700674 PMC3150044

[B25] Faith D. P. (1992). Conservation evaluation and phylogenetic diversity. *Biol. Conserv.* 61 1–10. 10.1016/0006-3207(92)91201-3

[B26] Fang W. St. Leger R. J. (2010). RNA binding proteins mediate the ability of a fungus to adapt to the cold. *Environ. Microbiol.* 12 810–820. 10.1111/j.1462-2920.2009.02127.x 20050869

[B27] Garrido-Jurado I. Torrent J. Barrón V. Corpas A. Quesada-Moraga E. (2011). Soil properties affect the availability, movement, and virulence of entomopathogenic fungi conidia against puparia of *Ceratitis capitata* (Diptera: Tephritidae). *Biol. Control* 58 277–285. 10.1016/j.biocontrol.2011.05.017

[B28] Genier H. L. A. Queiroz J. H. D. Braga F. R. Soares F. E. D. F. Araújo J. V. D. (2016). Nematicidal activity of *Paecilomyces marquandii* proteases on infective larvae of *Ancylostoma* spp. *Braz. Arch. Biol. Technol.* 59:e16160218.

[B29] Goble T. Dames J. Hill M. Moore S. (2010). The effects of farming system, habitat type and bait type on the isolation of entomopathogenic fungi from citrus soils in the Eastern Cape Province, South Africa. *BioControl* 55 399–412. 10.1007/s10526-009-9259-0

[B30] Gold A. Giraud T. Hood M. E. (2009). Within-host competitive exclusion among species of the anther smut pathogen. *BMC Ecol.* 9:11. 10.1186/1472-6785-9-11 19422703 PMC2688501

[B31] Güneralp B. Perlstein A. S. Seto K. C. (2015). Balancing urban growth and ecological conservation: a challenge for planning and governance in China. *Ambio* 44 532–543. 10.1007/s13280-015-0625-0 25640322 PMC4552716

[B32] Hajek A. E. Shapiro-Ilan D. I. (2018). *Ecology of Invertebrate Diseases.* Hoboken, NJ: John Wiley & Sons Ltd.

[B33] Hall T. A. (1999). BioEdit: a user-friendly biological sequence alignment editor and analysis program for Windows 95/98/NT. *Nucleic Acids Symp. Ser.* 41 95–98.

[B34] Hernández-Domínguez C. Guzmán-Franco A. W. (2017). Species diversity and population dynamics of entomopathogenic fungal species in the genus *Metarhizium*—a Spatiotemporal Study. *Microb. Ecol.* 74 194–206. 10.1007/s00248-017-0942-x 28124726

[B35] Hirsch J. Galidevara S. Strohmeier S. Devi K. U. Reineke A. (2013). Effects on diversity of soil fungal community and fate of an artificially applied *Beauveria bassiana* strain assessed through 454 pyrosequencing. *Microb. Ecol.* 66 608–620. 10.1007/s00248-013-0249-5 23736813

[B36] Humber R. A. (2012). “Identification of entomopathogenic fungi,” in *Manual of Techniques in Invertebrate Pathology*, ed. Lacey L. A. (London: Academic Press), 151–187. 10.1016/b978-0-12-386899-2.00006-3

[B37] Imoulan A. Wu H.-J. Lu W.-L. Li Y. Li B.-B. Yang R.-H. (2016). *Beauveria medogensis* sp. nov., a new fungus of the entomopathogenic genus from China. *J. Invertebr. Pathol.* 139 74–81. 10.1016/j.jip.2016.07.006 27449678

[B38] Inglis G. D. Duke G. M. Goettel M. S. Kabaluk J. T. (2008). Genetic diversity of *Metarhizium anisopliae* var. Anisopliae in southswestern British Columbia. *J. Invertebr. Pathol.* 98 101–113. 10.1016/j.jip.2007.12.001 18215399

[B39] Inglis G. D. Duke G. M. Goettel M. S. Kabaluk J. T. Ortega-Polo R. (2019). Biogeography and genotypic diversity of *Metarhizium brunneum* and *Metarhizium robertsii* in northwestern North American soils. *Can. J. Microbiol.* 65 261–281. 10.1139/cjm-2018-0297 30532988

[B40] Jaronski S. T. (2007). “Soil ecology of the entomopathogenic ascomycetes: a critical examination of what we (think) we know,” in *Use of Entomopathogenic Fungi in Biological Pest Management*, eds Ekesi S. Maniania N. K. (Trivandrum: Research Signpost), 91–143.

[B41] Jaronski S. T. (2010). Ecological factors in the inundative use of fungal entomopathogens. *BioControl* 55 159–185. 10.1007/978-90-481-3966-8_12

[B42] Katoh K. Standley D. M. (2013). MAFFT multiple sequence alignment software version 7: improvements in performance and usability. *Mol. Biol. Evol.* 30 772–780. 10.1093/molbev/mst010 23329690 PMC3603318

[B43] Keller S. Kessler P. Schweizer C. (2003). Distribution of insect pathogenic soil fungi in Switzerland with special reference to *Beauveria brongniartii* and *Metharhizium anisopliae*. *BioControl* 48 307–319.

[B44] Kepler R. Ban S. Nakagiri A. Bischoff J. Hywel-Jones N. Owensby C. A. (2013). The phylogenetic placement of hypocrealean insect pathogens in the genus *Polycephalomyces*: an application of one fungus one name. *Fungal Biol.* 117 611–622. 10.1016/j.funbio.2013.06.002 24012301

[B45] Kepler R. M. Humber R. A. Bischoff J. F. Rehner S. A. (2014). Clarification of generic and species boundaries for *Metarhizium* and related fungi through multigene phylogenetics. *Mycologia* 106 811–829. 10.3852/13-319 24891418

[B46] Kepler R. M. Rehner S. A. (2013). Genome-assisted development of nuclear intergenic sequence markers for entomopathogenic fungi of the *Metarhizium anisopliae* species complex. *Mol. Ecol. Resour.* 13 210–217. 10.1111/1755-0998.12058 23286460

[B47] Kepler R. M. Sung G. H. Ban S. Nakagiri A. Chen M. J. Huang B. (2012). New teleomorph combinations in the entomopathogenic genus *Metacordyceps*. *Mycologia* 104 182–197. 10.3852/11-070 22067304

[B48] Kepler R. M. Ugine T. A. Maul J. E. Cavigelli M. A. Rehner S. A. (2015). Community composition and population genetics of insect pathogenic fungi in the genus *Metarhizium* from soils of a long-term agricultural research system. *Environ. Microbiol.* 17 2791–2804. 10.1111/1462-2920.12778 25627647

[B49] Keyser C. A. De Fine Licht H. H. Steinwender B. M. Meyling N. V. (2015). Diversity within the entomopathogenic fungal species *Metarhizium flavoviride* associated with agricultural crops in Denmark. *BMC Microbiol.* 15:249. 10.1186/s12866-015-0589-z 26519342 PMC4628438

[B50] Kirk P. Canon P. Minter D. Staplers J. (2008). *Dictionary of the Fungi.* Wallingford: CABI.

[B51] Klingen I. Eilenberg J. Meadow R. (2002). Effects of farming system, field margins and bait insect on the occurrence of insect pathogenic fungi in soils. *Agric. Ecosyst. Environ.* 91 191–198. 10.1016/s0167-8809(01)00227-4

[B52] Kõljalg U. Nilsson R. H. Abarenkov K. Tedersoo L. Taylor A. F. S. Bahram M. (2013). Towards a unified paradigm for sequence-based identification of fungi. *Mol. Ecol.* 22 5271–5277. 24112409 10.1111/mec.12481

[B53] Korosi G. A. Wilson B. A. L. Powell K. S. Ash G. J. Reineke A. Savocchia S. (2019). Occurrence and diversity of entomopathogenic fungi (*Beauveria* spp. and *Metarhizium* spp.) in Australian vineyard soils. *J. Invertebr. Pathol.* 164 69–77. 10.1016/j.jip.2019.05.002 31078548

[B54] Kruskal W. H. Wallis W. A. (1952). Use of ranks in one-criterion variance analysis. *J. Am. Stat. Assoc.* 47 583–621. 10.1080/01621459.1952.10483441

[B55] Kumar S. Stecher G. Tamura K. (2016). MEGA7: molecular evolutionary genetics analysis version 7.0 for bigger datasets. *Mol. Biol. Evol.* 33 1870–1874. 10.1093/molbev/msw054 27004904 PMC8210823

[B56] Legendre P. Anderson M. J. (1999). Distance-based redundancy analysis: testing multispecies responses in multifactorial ecological experiments. *Ecol. Monogr.* 69 1–24. 10.1890/0012-9615(1999)069[0001:dbratm]2.0.co;2

[B57] Lozupone C. A. Hamady M. Kelley S. T. Knight R. (2007). Quantitative and qualitative β diversity measures lead to different insights into factors that structure microbial communities. *Appl. Environ. Microbiol.* 73 1576–1585. 10.1128/aem.01996-06 17220268 PMC1828774

[B58] Luz C. Rocha L. F. N. Montalva C. Souza D. A. Beatriz R. Z. Botelho A. (2019). *Metarhizium humberi* sp. nov. (Hypocreales: Clavicipitaceae), a new member of the PARB clade in the *Metarhizium anisopliae* complex from Latin America. *J. Invertebr. Pathol.* 166:107216. 10.1016/j.jip.2019.107216 31299226

[B59] Maciá-Vicente J. G. Rosso L. C. Ciancio A. Jansson H.-B. Lopez-Llorca L. V. (2009). Colonisation of barley roots by endophytic *Fusarium equiseti* and *Pochonia chlamydosporia*: effects on plant growth and disease. *Ann. Appl. Biol.* 155 391–401. 10.1111/j.1744-7348.2009.00352.x

[B60] Malacrinò A. Schena L. Campolo O. Laudani F. Mosca S. Giunti G. (2017). A metabarcoding survey on the fungal microbiota associated to the olive fruit fly. *Microb. Ecol.* 73 677–684. 10.1007/s00248-016-0864-z 27687872

[B61] Martin M. (2011). Cutadapt removes adapter sequences from high-throughput sequencing reads. *EMBnet J.* 17 10–12.

[B62] Masoudi A. Koprowski J. L. Bhattarai U. R. Wang D. (2018). Elevational distribution and morphological attributes of the entomopathogenic fungi from forests of the Qinling Mountains in China. *Appl. Microbiol. Biotechnol.* 102 1483–1499. 10.1007/s00253-017-8651-4 29189901

[B63] McKinnon A. C. Glare T. R. Ridgway H. J. Mendoza-Mendoza A. Holyoake A. Godsoe W. K. (2018). Detection of the entomopathogenic fungus *Beauveria bassiana* in the rhizosphere of wound-stressed *Zea mays* plants. *Front. Microbiol.* 9:1161. 10.3389/fmicb.2018.01161 29942287 PMC6004820

[B64] McKinnon A. C. Saari S. Moran-Diez M. E. Meyling N. V. Raad M. Glare T. R. (2017). *Beauveria bassiana* as an endophyte: a critical review on associated methodology and biocontrol potential. *BioControl* 62 1–17. 10.1007/s10526-016-9769-5

[B65] Medo J. Cagáň L’ (2011). Factors affecting the occurrence of entomopathogenic fungi in soils of Slovakia as revealed using two methods. *Biol. Control* 59 200–208. 10.1016/j.biocontrol.2011.07.020

[B66] Meyling N. V. (2007). *Methods for Isolation of Entomopathogenic Fungi from the Soil Environment.* Copenhagen: University of Copenhagen.

[B67] Meyling N. V. Eilenberg J. (2006). Occurrence and distribution of soil borne entomopathogenic fungi within a single organic agroecosystem. *Agric. Ecosyst. Environ.* 113 336–341. 10.1016/j.agee.2005.10.011

[B68] Meyling N. V. Lübeck M. Buckley E. P. Eilenberg J. Rehner S. A. (2009). Community composition, host range and genetic structure of the fungal entomopathogen *Beauveria* in adjoining agricultural and seminatural habitats. *Mol. Ecol.* 18 1282–1293. 10.1111/j.1365-294x.2009.04095.x 19226319

[B69] Meyling N. V. Pilz C. Keller S. Widmer F. Enkerli J. (2012). Diversity of *Beauveria* spp. isolates from pollen beetles *Meligethes aeneus* in Switzerland. *J. Invertebr. Pathol.* 109 76–82. 10.1016/j.jip.2011.10.001 22008375

[B70] Meyling N. V. Thorup-Kristensen K. Eilenberg J. (2011). Below- and aboveground abundance and distribution of fungal entomopathogens in experimental conventional and organic cropping systems. *Biol. Control* 59 180–186. 10.1016/j.biocontrol.2011.07.017

[B71] Mietkiewski R. Tkaczuk C. (1998). The spectrum and frequency of entomopathogenic fungi in litter, forest soil and arable soil. *IOBC/WPRS Bull.* 21 41–44.

[B72] Miller M. A. Pfeiffer W. Schwartz T. (2010). “Creating the CIPRES Science Gateway for inference of large phylogenetic trees,” in *Proceedings of the 2010 Gateway Computing Environments Workshop (GCE)*), New Orleans, LA, 1–8.

[B73] Moonjely S. Bidochka M. J. (2019). Generalist and specialist *Metarhizium* insect pathogens retain ancestral ability to colonize plant roots. *Fungal Ecol.* 41 209–217. 10.1016/j.funeco.2019.06.004

[B74] Oddsdóttir E. S. Nielsen C. Sen R. Harding S. Eilenberg J. Halldórsson G. (2010). Distribution patterns of soil entomopathogenic and birch symbiotic ectomycorrhizal fungi across native woodland and degraded habitats in Iceland. *Icelandic Agric. Sci.* 32 37–49.

[B75] Oksanen J. Blanchet F. Kindt R. Legendre P. Minchin P. O’hara R. (2013). *Package ‘Vegan’. Community Ecology Package, Version* 2. Available online at: http://cran.rproject.org/web/packages/vegan/index.html (accessed February 12, 2019).

[B76] Ondov B. D. Bergman N. H. Phillippy A. M. (2011). Interactive metagenomic visualization in a web browser. *BMC Bioinformatics* 12:385. 10.1186/1471-2105-12-385 21961884 PMC3190407

[B77] Orgiazzi A. Lumini E. Nilsson R. H. Girlanda M. Vizzini A. Bonfante P. (2012). Unravelling soil fungal communities from different mediterranean land-use backgrounds. *PLoS One* 7:e34847. 10.1371/journal.pone.0034847 22536336 PMC3335027

[B78] Ottosson E. Kubartová A. Edman M. Jönsson M. Lindhe A. Stenlid J. (2015). Diverse ecological roles within fungal communities in decomposing logs of *Picea abies*. *FEMS Microbiol.Ecol.* 91:fiv012. 10.1093/femsec/fiv012 25764460

[B79] Pattemore J. A. Hane J. K. Williams A. H. Wilson B. A. Stodart B. J. Ash G. J. (2014). The genome sequence of the biocontrol fungus *Metarhizium anisopliae* and comparative genomics of *Metarhizium* species. *BMC Genomics* 15:660. 10.1186/1471-2164-15-660 25102932 PMC4133081

[B80] Pearce D. A. Bridge P. D. Hughes K. A. Sattler B. Psenner R. Russell N. J. (2009). Microorganisms in the atmosphere over Antarctica. *FEMS Microbiol. Ecol.* 69 143–157. 10.1111/j.1574-6941.2009.00706.x 19527292

[B81] Pirali-Kheirabadi K. Haddadzadeh H. Razzaghi-Abyaneh M. Bokaie S. Zare R. Ghazavi M. (2007). Biological control of *Rhipicephalus* (Boophilus) annulatus by different strains of *Metarhizium anisopliae*, *Beauveria bassiana* and *Lecanicillium psalliotae* fungi. *Parasitol. Res.* 100 1297–1302. 10.1007/s00436-006-0410-x 17186273

[B82] Qu J. Zhou Y. Yu J. Zhang J. Han Y. Zou X. (2018). Estimated divergence times of *Hirsutella* (asexual morphs) in *Ophiocordyceps* provides insight into evolution of phialide structure. *BMC Evol. Biol.* 18:111. 10.1186/s12862-018-1223-0 30005592 PMC6043951

[B83] Quesada-Moraga E. Navas-Cortés J. A. Maranhao E. A. Ortiz-Urquiza A. Santiago-Álvarez C. (2007). Factors affecting the occurrence and distribution of entomopathogenic fungi in natural and cultivated soils. *Mycol. Res.* 111 947–966. 10.1016/j.mycres.2007.06.006 17766099

[B84] R Core Team (2016). *R: A Language and Environment for Statistical Computing*. Vienna: R Foundation for Statistical Computing Available online at: http://www.R-project.org/

[B85] Ramos Y. Portal O. Lysøe E. Meyling N. V. Klingen I. (2017). Diversity and abundance of *Beauveria bassiana* in soils, stink bugs and plant tissues of common bean from organic and conventional fields. *J. Invertebr. Pathol.* 150 114–120. 10.1016/j.jip.2017.10.003 29042323

[B86] Reeder J. Knight R. (2010). Rapidly denoising pyrosequencing amplicon reads by exploiting rank-abundance distributions. *Nat. Methods* 7 668–669. 10.1038/nmeth0910-668b 20805793 PMC2945879

[B87] Rehner S. A. Kepler R. M. (2017). Species limits, phylogeography and reproductive mode in the *Metarhizium anisopliae* complex. *J. Invertebr. Pathol.* 148 60–66. 10.1016/j.jip.2017.05.008 28578154

[B88] Rehner S. A. Minnis A. M. Sung G.-H. Luangsa-Ard J. J. Devotto L. Humber R. A. (2011). Phylogeny and systematics of the anamorphic, entomopathogenic genus *Beauveria*. *Mycologia* 103 1055–1073. 10.3852/10-302 21482632

[B89] Rezende J. M. Zanardo A. B. R. Da Silva Lopes M. Delalibera I. Rehner S. A. (2015). Phylogenetic diversity of Brazilian *Metarhizium* associated with sugarcane agriculture. *BioControl* 60 495–505. 10.1007/s10526-015-9656-5

[B90] Robène-Soustrade I. Jouen E. Pastou D. Payet-Hoarau M. Goble T. Linderme D. (2015). Description and phylogenetic placement of *Beauveria hoplocheli* sp. nov. used in the biological control of the sugarcane white grub, *Hoplochelus marginalis*, on Reunion Island. *Mycologia* 107 1221–1232. 10.3852/14-344 26297783

[B91] Rocha L. F. N. Inglis P. W. Humber R. A. Kipnis A. Luz C. (2013). Occurrence of *Metarhizium* spp. in Central Brazilian soils. *J. Basic Microbiol.* 53 251–259. 10.1002/jobm.201100482 22733433

[B92] Scheepmaker J. W. A. Butt T. M. (2010). Natural and released inoculum levels of entomopathogenic fungal biocontrol agents in soil in relation to risk assessment and in accordance with EU regulations. *Biocontrol Sci. Technol.* 20 503–552. 10.1080/09583150903545035

[B93] Segata N. Izard J. Waldron L. Gevers D. Miropolsky L. Garrett W. S. (2011). Metagenomic biomarker discovery and explanation. *Genome Biol.* 12:R60. 10.1186/gb-2011-12-6-r60 PMC3218848 21702898

[B94] Serna-Domínguez M. G. Andrade-Michel G. Y. Arredondo-Bernal H. C. Gallou A. (2018). Two efficient methods for isolation of high-quality genomic DNA from entomopathogenic fungi. *J. Microbiol. Methods* 148 55–63. 10.1016/j.mimet.2018.03.012 29596959

[B95] Shade A. Dunn R. R. Blowes S. A. Keil P. Bohannan B. J. M. Herrmann M. (2018). Macroecology to unite all life, large and small. *Trends Ecol. Evol.* 33 731–744. 10.1016/j.tree.2018.08.005 30209011

[B96] Shannon C. E. (1948). A mathematical theory of communication. *Bell Syst. Tech. J.* 27 379–423.

[B97] Simmons D. R. Kepler R. M. Renner S. A. Groden E. (2015). Phylogeny of *Hirsutella* species (Ophiocordycipitaceae) from the USA: remedying the paucity of *Hirsutella* sequence data. *IMA Fungus* 6 345–356. 10.5598/imafungus.2015.06.02.06 26734545 PMC4681258

[B98] Simpson E. H. (1949). Measurement of diversity. *Nature* 163:688.

[B99] Stamatakis A. (2014). RAxML version 8: a tool for phylogenetic analysis and post-analysis of large phylogenies. *Bioinformatics* 30 1312–1313. 10.1093/bioinformatics/btu033 24451623 PMC3998144

[B100] Steinwender B. M. Enkerli J. Widmer F. Eilenberg J. Kristensen H. L. Bidochka M. J. (2015). Root isolations of *Metarhizium* spp. from crops reflect diversity in the soil and indicate no plant specificity. *J. Invertebr. Pathol.* 132 142–148. 10.1016/j.jip.2015.09.007 26407950

[B101] Steinwender B. M. Enkerli J. Widmer F. Eilenberg J. Thorup-Kristensen K. Meyling N. V. (2014). Molecular diversity of the entomopathogenic fungal *Metarhizium* community within an agroecosystem. *J. Invertebr. Pathol.* 123 6–12. 10.1016/j.jip.2014.09.002 25224815

[B102] Sun B.-D. Yu H.-Y. Chen A. J. Liu X.-Z. (2008). Insect-associated fungi in soils of field crops and orchards. *Crop Prot.* 27 1421–1426. 10.1016/j.cropro.2008.07.010

[B103] Tall S. Meyling N. V. (2018). Probiotics for plants? growth promotion by the entomopathogenic fungus *Beauveria bassiana* depends on nutrient availability. *Microb. Ecol.* 76 1002–1008. 10.1007/s00248-018-1180-6 29594431

[B104] Tigano M. S. Adams B. Maimala S. Boucias D. (2006). Genetic diversity of *Hirsutella thompsonii* isolates from Thailand based on AFLP analysis and partial b-tubulin gene sequences. *Genet. Mol. Biol.* 29 715–721. 10.1590/s1415-47572006000400022

[B105] Tremblay ÉD. Kimoto T. Bérubé J. A. Bilodeau G. J. (2019). High-throughput sequencing to investigate phytopathogenic fungal propagules caught in baited insect traps. *J. Fungi* 5:15. 10.3390/jof5010015 30759800 PMC6463110

[B106] Treschow C. (1941). The *Verticillium* diseases of cultivated Mushrooms. *Dan. Bot. Ark.* 11 1–31.

[B107] Vänninen I. (1996). Distribution and occurrence of four entomopathogenic fungi in Finland: effect of geographical location, habitat type and soil type. *Mycol. Res.* 100 93–101. 10.1016/s0953-7562(96)80106-7

[B108] Vega F. E. Kaya H. K. (2012). *Insect Pathology.* San Diego, CA: Academic press.

[B109] Wakil W. Ghazanfar M. U. Riasat T. Kwon Y. J. Qayyum M. A. Yasin M. (2013). Occurrence and diversity of entomopathogenic fungi in cultivated and uncultivated soils in Pakistan. *Entomol. Res.* 43 70–78. 10.1111/1748-5967.12003

[B110] Wang J. B. St. Leger R. J. Wang C. (2016). “Chapter Three - Advances in Genomics of Entomopathogenic Fungi,” in *Advances in Genetics*, eds Lovett B. St. Leger R. J. (San Diego, CA: Academic Press), 67–105. 10.1016/bs.adgen.2016.01.002 27131323

[B111] Wyrebek M. Huber C. Sasan R. K. Bidochka M. J. (2011). Three sympatrically occurring species of *Metarhizium* show plant rhizosphere specificity. *Microbiology* 157 2904–2911. 10.1099/mic.0.051102-0 21778205

[B112] Yang Y. Zhao M. Xu X. Sun Z. Yin G. Piao S. (2014). Diurnal and Seasonal Change in Stem Respiration of *Larix principis*-*rupprechtii* Trees, Northern China. *PLoS One* 9:e89294. 10.1371/journal.pone.0089294 24586668 PMC3935864

[B113] Zare R. Gams W. (2001). A revision of *Verticillium* section *Prostrat*a. IV. The genera *Lecanicillium* and *Simplicillium* gen. nov. *Nova Hedwigia* 3 1–50.

[B114] Zhang S.-L. He L.-M. Chen X. Huang B. (2013). *Beauveria lii* sp. nov. isolated from *Henosepilachna vigintioctopunctata*. *Mycotaxon* 121 199–206. 10.5248/121.199

[B115] Zimmermann G. (1986). The ‘*Galleria* bait method’for detection of entomopathogenic fungi in soil. *J. Appl. Entomol.* 102 213–215. 10.1111/j.1439-0418.1986.tb00912.x

