# Supplementary material for: Meta-Analysis and Evaluation by Insect-Mediated Baiting Reveal Different Patterns of Hypocrealean Entomopathogenic Fungi in the Soils From Two Regions of China
Source: Front Microbiol. 2020 Jun 12;11:1133. doi: 10.3389/fmicb.2020.01133 (PMC7303310; doi:10.3389/fmicb.2020.01133)
Supplement: Supplementary file 1 [file Data_Sheet_1.zip › Supplementary Figures.DOCX]

**Meta-Analysis and Evaluation by Insect-Mediated Baiting Reveal Different Patterns of Hypocrealean Entomopathogenic Fungi in the Soils From Two Regions of China**

Abolfazl Masoudi, Min Wang, Xiaoli Zhang, Can Wang, Zhaoxi Qiu, Wenying Wang, Hui Wang^*^, Jingze Liu^**^

Hebei Key Laboratory of Animal Physiology, Biochemistry and Molecular biology, College of Life Sciences, Hebei Normal University, Shijiazhuang 050024, China

Present address: 20 Nanerhuan East Road, Shijiazhuang, Hebei Province

To whom correspondence should be addressed: *Corresponding Author: Tel/fax: +86 311 80787551, [whui1981@163.com](mailto:whui1981@163.com) (H. Wang); **Corresponding Author: Tel/fax: +86 311 80787552, [liujingze@hebtu.edu.cn](mailto:liujingze@hebtu.edu.cn) (J. Liu).

Keywords: High-throughput sequencing, Entomopathogenic soil fungi, Baiting method, *Metarhizium*, *Beauveria*, Mating-type


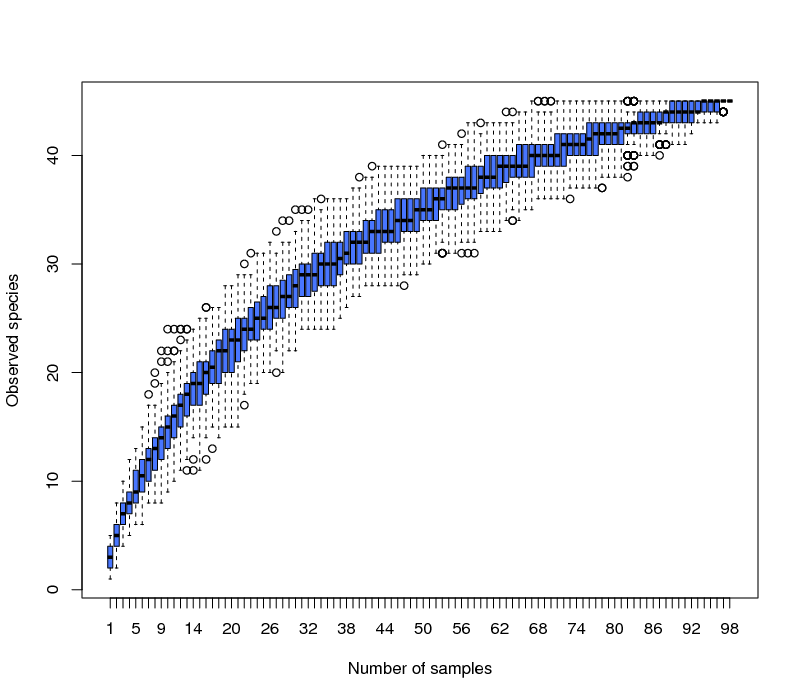


Supplementary Figure S1: Species accumulation cure (Specaccum) for EFs communities from the 98 sampling sites.


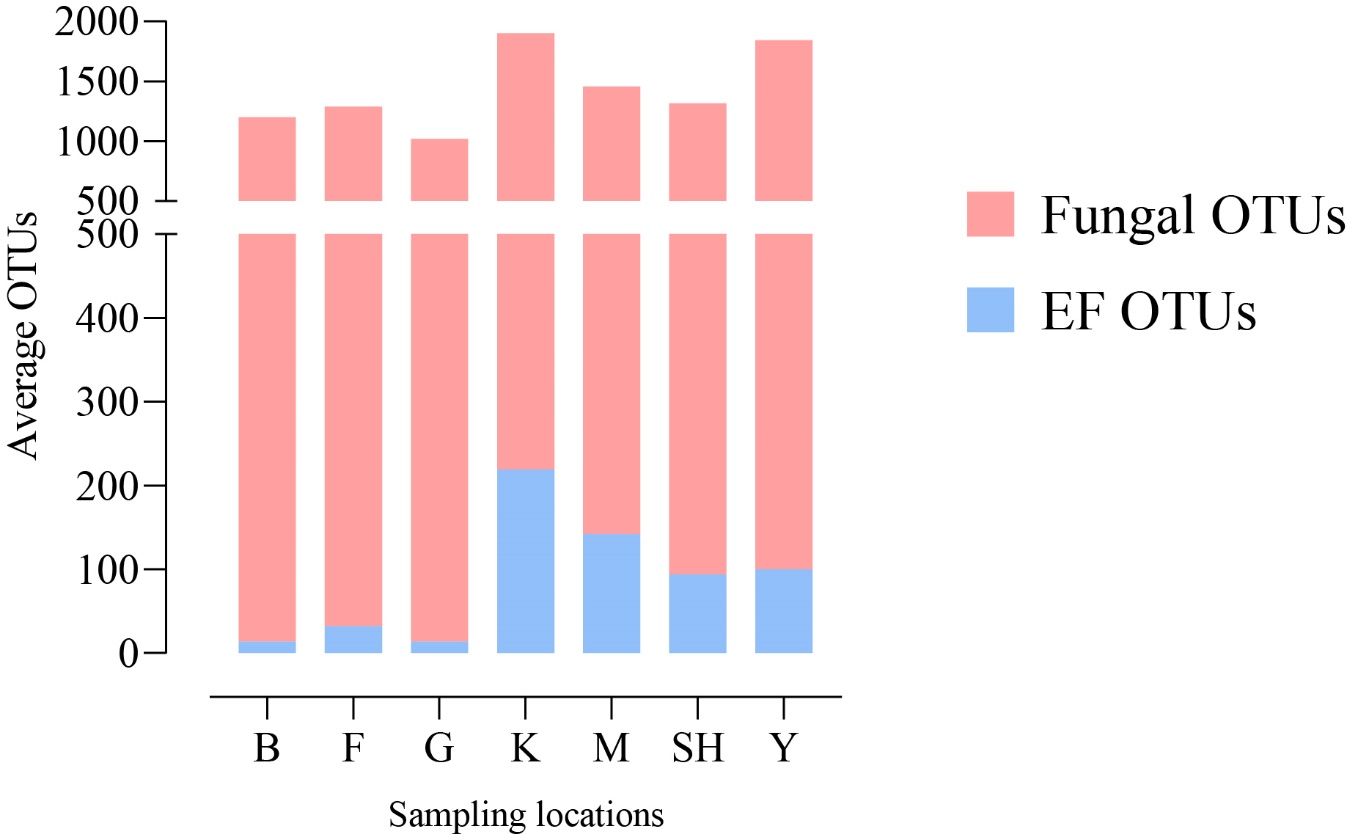


Supplementary Figure S2: The proportion of the EF OTUs in comparison with the whole fungal OTU compositions.


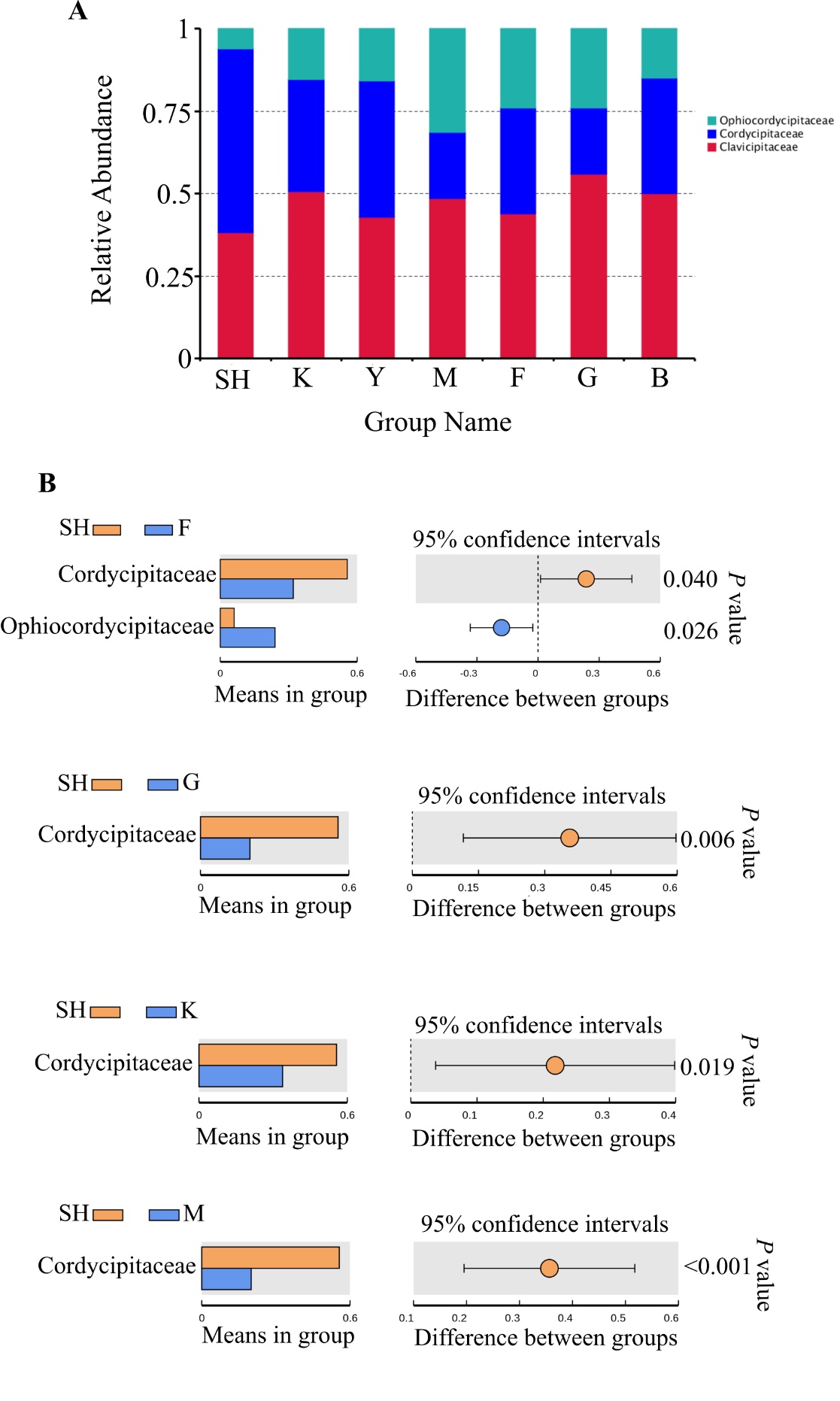


Supplementary Figure S3: This picture shows a relative abundance of the three EFs families at 7 collection sites (A), and *t*-test displays the significant level statistically based on the pairwise comparisons at the sampling locations (B).


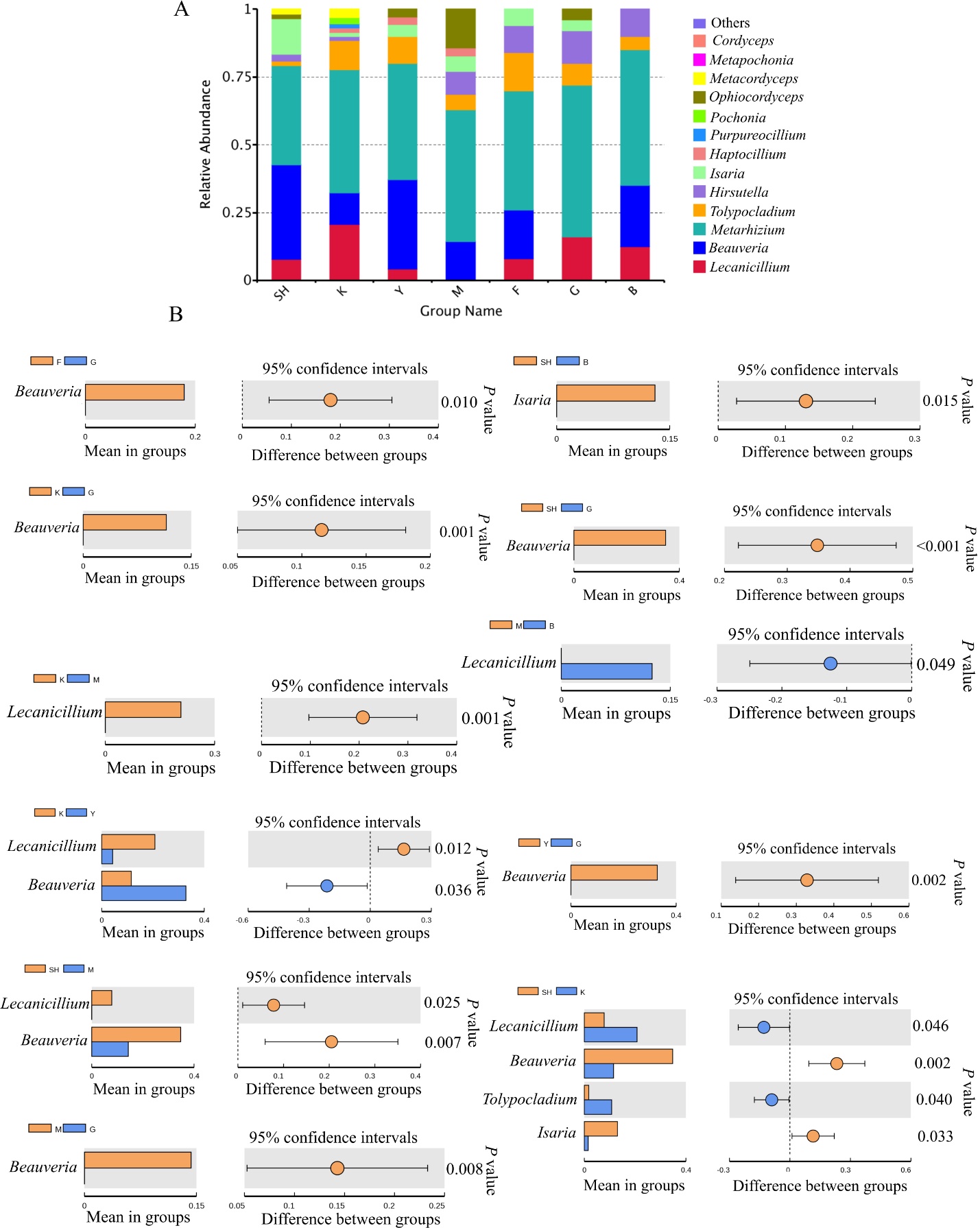


**A**

**B**


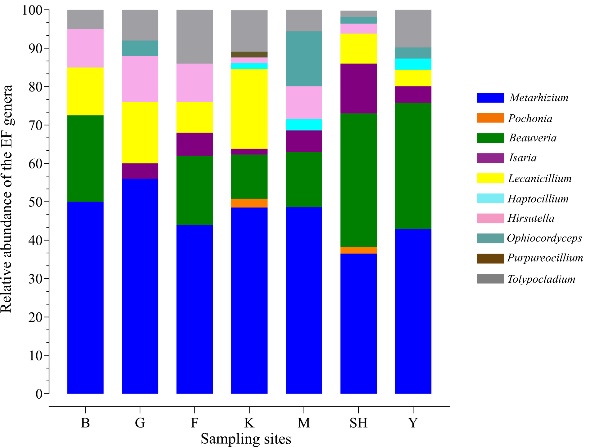


Supplementary Figure S4: This picture shows a relative abundance of top 10 of the EFs genera at the 7 collection sites (A), and *t*-test displays the significant level statistically based on the pairwise comparisons at the sampling locations (B).


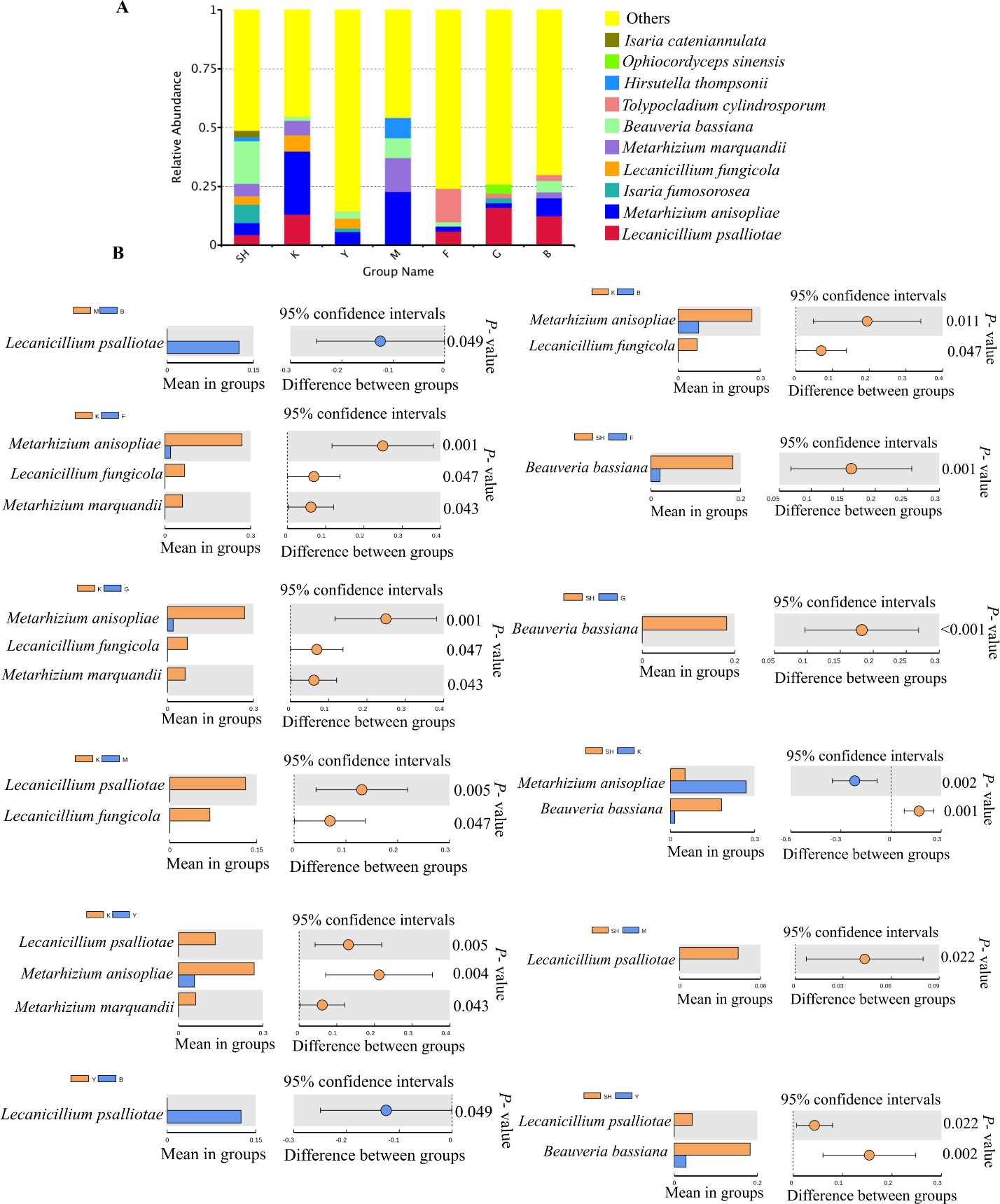


Supplementary Figure S5: This picture shows a relative abundance of top 10 of the EFs species at the 7 collection sites (A), and *t*-test displays the significant level statistically based on the pairwise comparisons at the sampling locations.


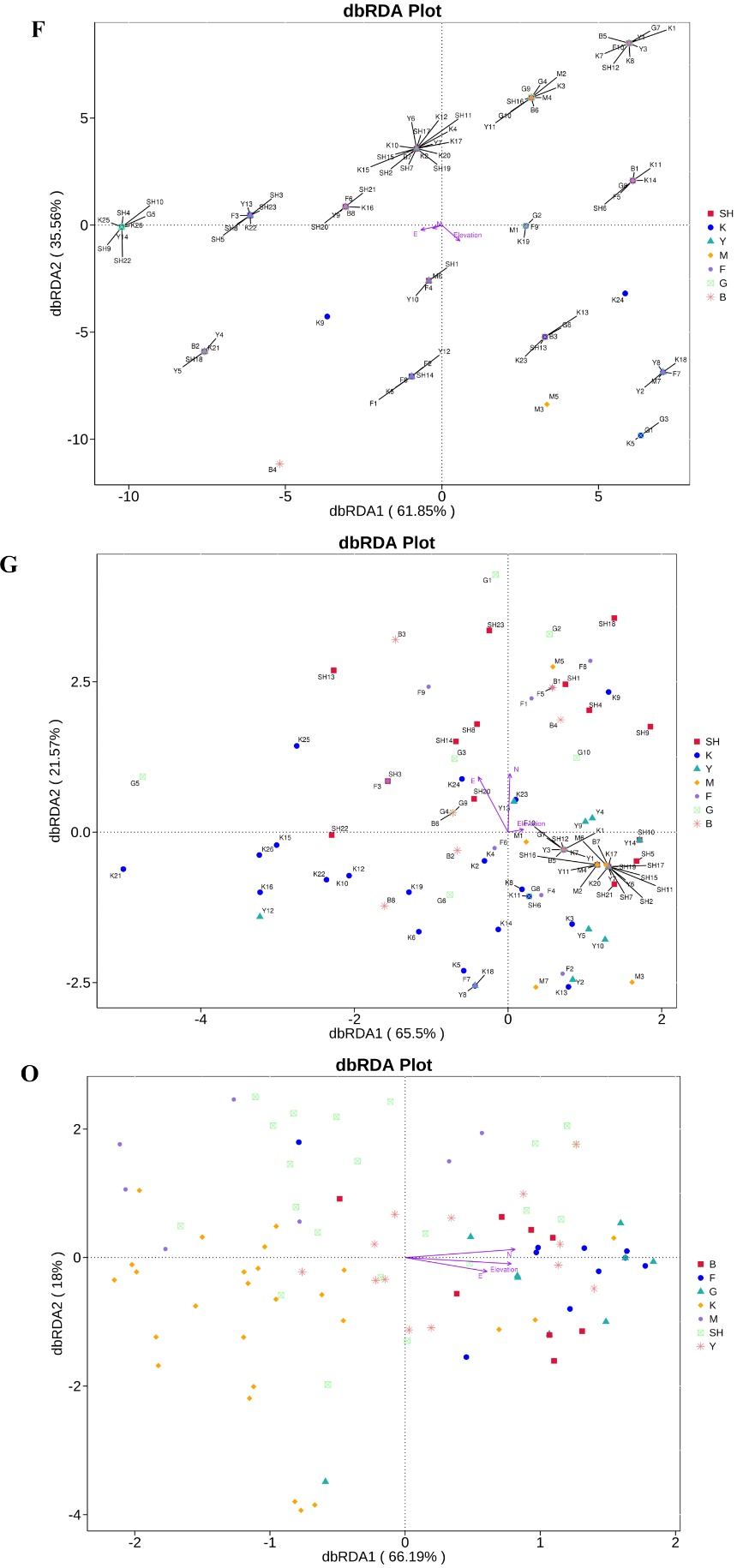


Supplementary Figure S6: This picture is illustrating the relationship between geographical coordinates at the Family (F), genus (G) and OTUs (O) levels.


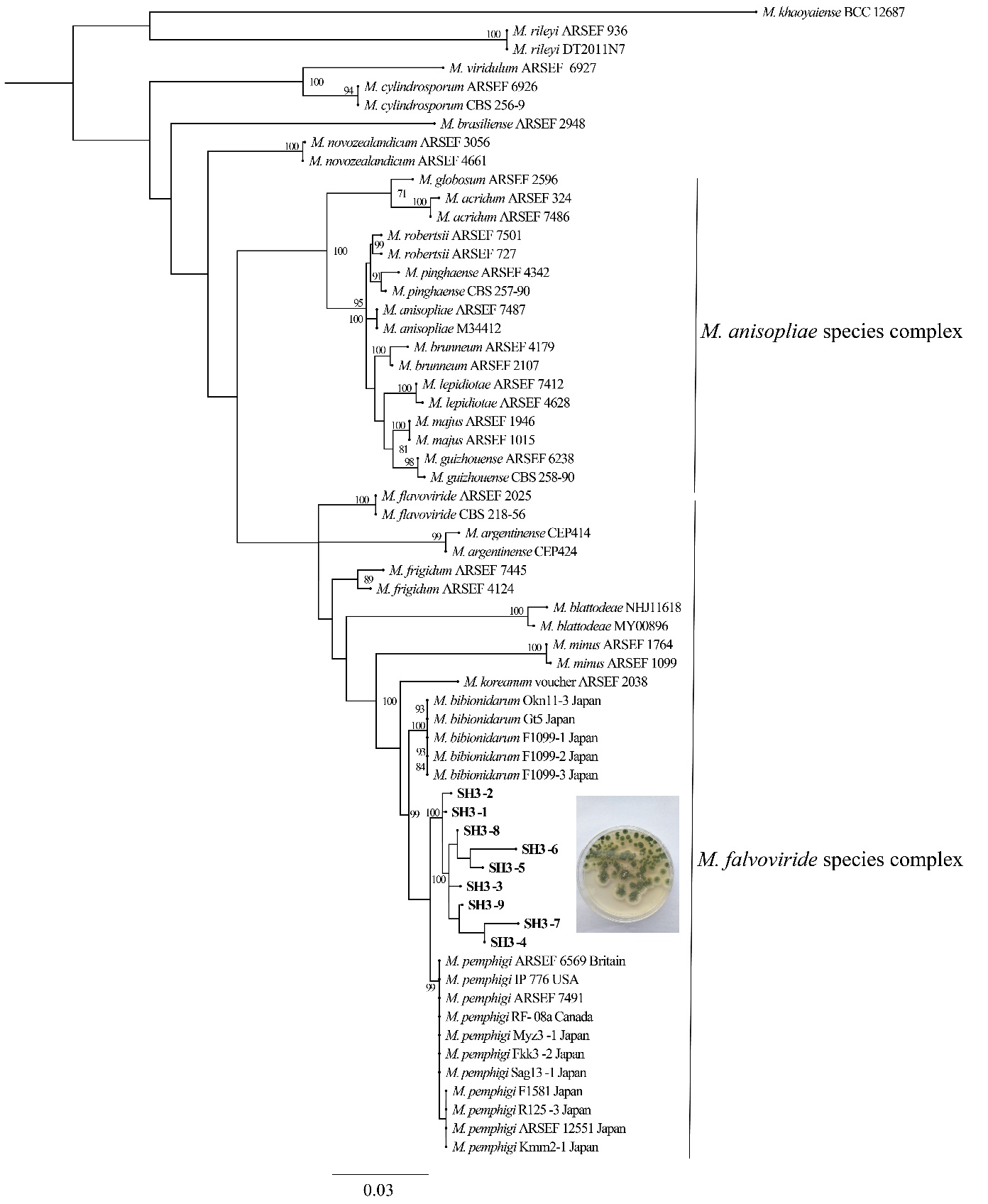


**Supplemental Figure S7**: RaxML 5TEF phylogeny of the 9 indigenous Chinese *Metarhizium* isolates within the *M. flavoviride* species complex. Bootstrap values ≥ 70% provided above relevant branches. ARSEF (USDA-ARS Collection of Entomopathogenic Fungal Cultures, Ithaca, NY, USA), CBS (Centraalbureau voor schimmelcultures Fungal Biodiversity Center, Utrecht, the Netherlands). All the reference sequences were obtained from Bischoff et al. (2009) and Kepler et al. (2014).


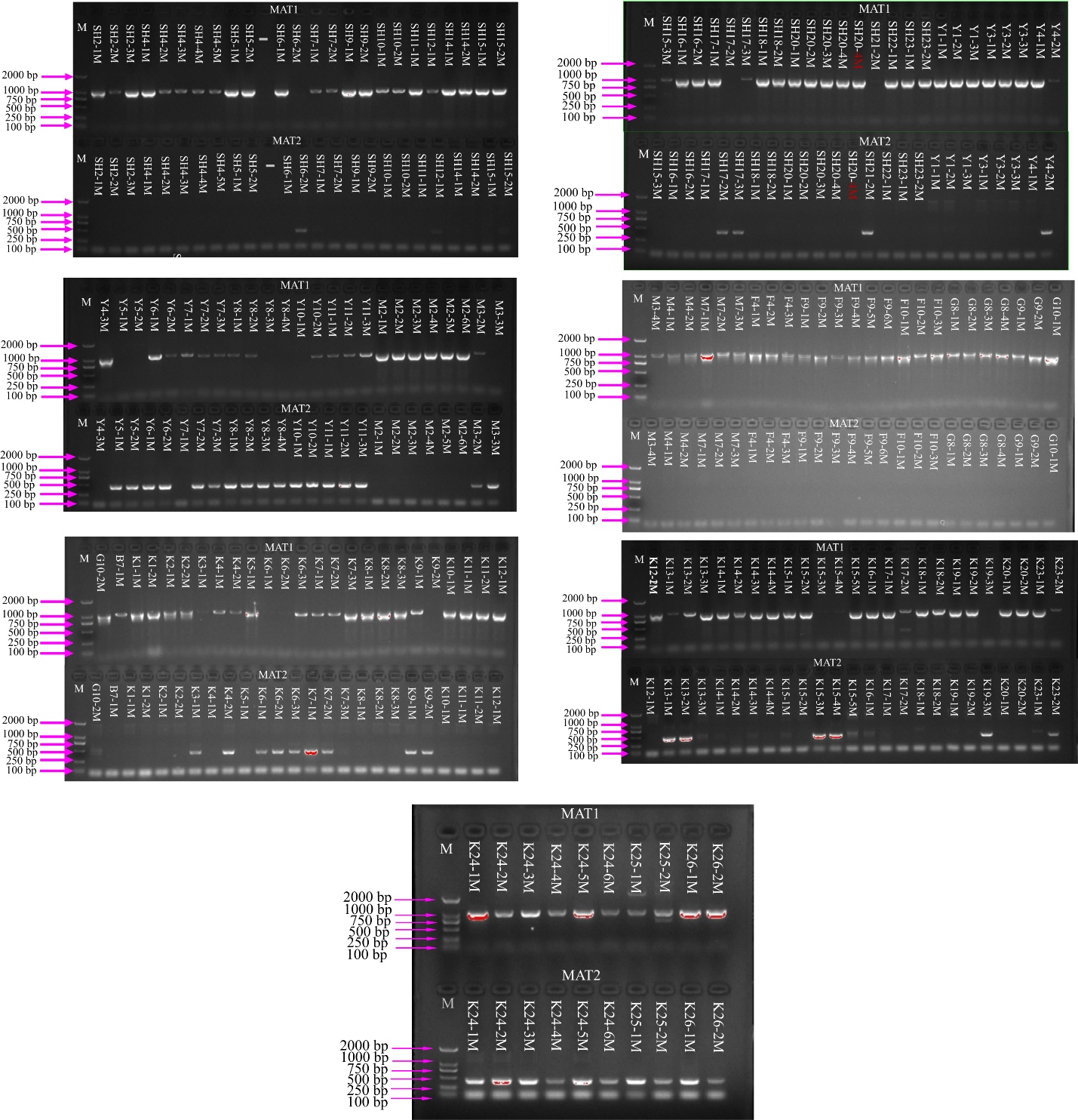


Supplementary Figure S8: mating-type determination of the *Metarhizium* isolates by PCR.

Supplementary Figure S9: mating-type determination of the *Beauveria* isolates by PCR.


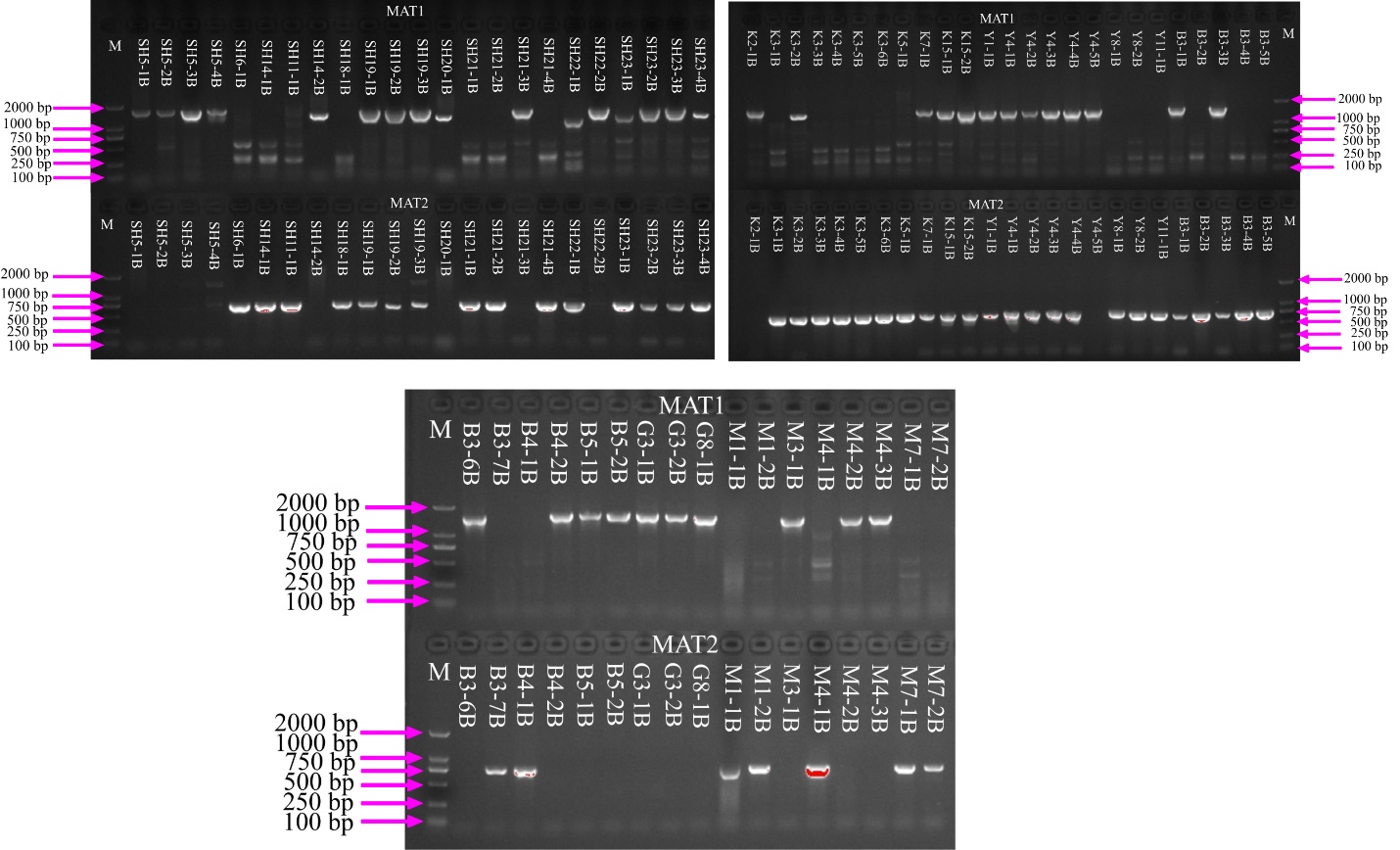


**The brief information on PCR diagnosis of *Beauveria* and *Metarhizium* mating-type**

Internal segments of MAT1-1-1 and MAT1-1-2 mating-type idiomorphs were analyzed by PCR. For *Beauveria*, MAT1 specific primers MAT112.F4 (5'-CAG CTC TCC GTC TGC CGA GTT-3′)/MAT111.R5 (5′-TAG TGA GAA AGC CTG ACG CGG-3′) and MAT2 specific primers MAT2.F4 (5'-RTC AGC GTC GGC ATC AAC CCA TT-3')/MAT2.R5 (5'-GAA AAY TCG CTG CCA GTC ATR AT-3') were used. PCR amplifications were conducted using the single thermal cycling profile that consisted of the single cycle of 95 °C for 2 min followed by 40 cycles of 95 °C for the 30s, 56 °C for 30s, 72 °C for 1 min and concluding with a single cycle of 72 °C for 15 min (Meyling et al., 2009). For *Metarhizium*, a 991 bp fragment of the MAT1-1-1 idiomorph was amplified using the primer pairs MAT111_1F (5'-TGG CGA CTC GGG CAG AAC TCA-3') and MAT111_3R (5'-GTT GGT GCA CCG GCA AGC GA-3') using a single cycle of 95 °C for 2 min followed by 36 cycles of 95 °C for 30s, 68 °C for 30 s, 72 °C for 1 min and concluding with a single cycle of 72 °C for 15 min. A 436 bp portion of the MAT1-2-1 idiomorph was amplified using the primers MAT121_3F (5'-ACC CAG CGG CTC GGA TCC AT-3') and MAT121_4R (5'-TCC AGT AGC CGA GTT CAT-3') using a touchdown procedure with an initial denaturing step for 30s at 95 °C, followed by 10 touchdown annealing cycles of 30 s that decreased the annealing temperature one degree every cycle for 10 cycles, followed by 40 cycles with annealing at 54 °C for 30 s and 72 °C extension for 1 min, concluding with a final extension at 72 °C for 10 min (Kepler et al., 2015; Kepler et al., 2016; Rehner and Kepler, 2017). PCR amplifications were conducted separately for each MAT primer pair, and their products resolved on 1.5 % agarose gels.


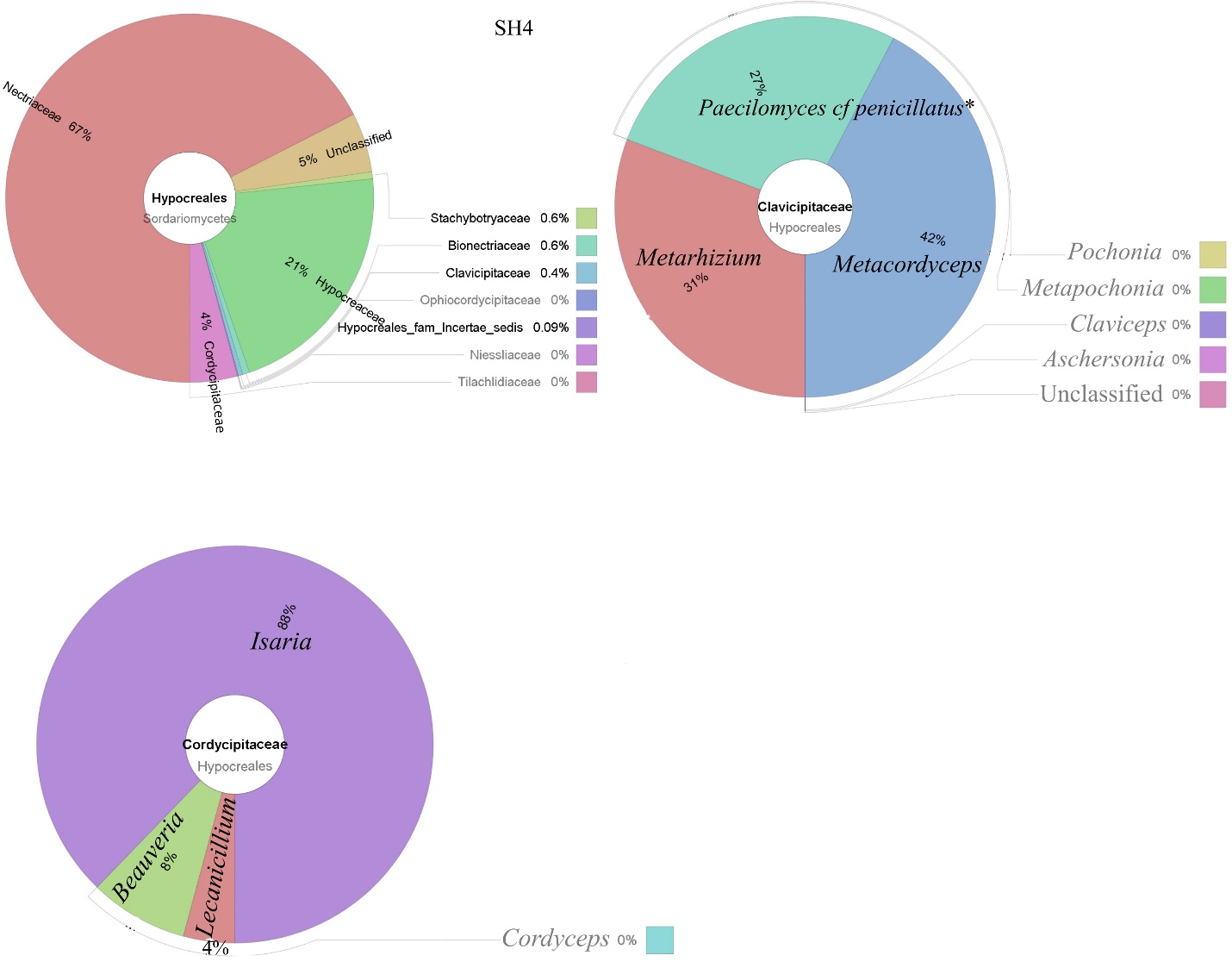


Supplementary Figure S10: Taxonomic composition of EFs in the soil samples of the one sites: SH4. * *Paecillomyces* cf *penicillatus* species does not have clear taxonomic information. Jennifer Luangsa-ard et al. (2011) showed that *P*. cf *penicillatus* was classified to Hypocreaceae family, but the information avalibility on data bases showed this species is belonged to Clavicipitaceae. However, Mycobank information (http://www.mycobank.org/name/Paecilomyces%20penicillatus&Lang=Eng) classified this genus to Trichocomaceae family. For this reason, we do not consider the frequency of this species in our main analysis.


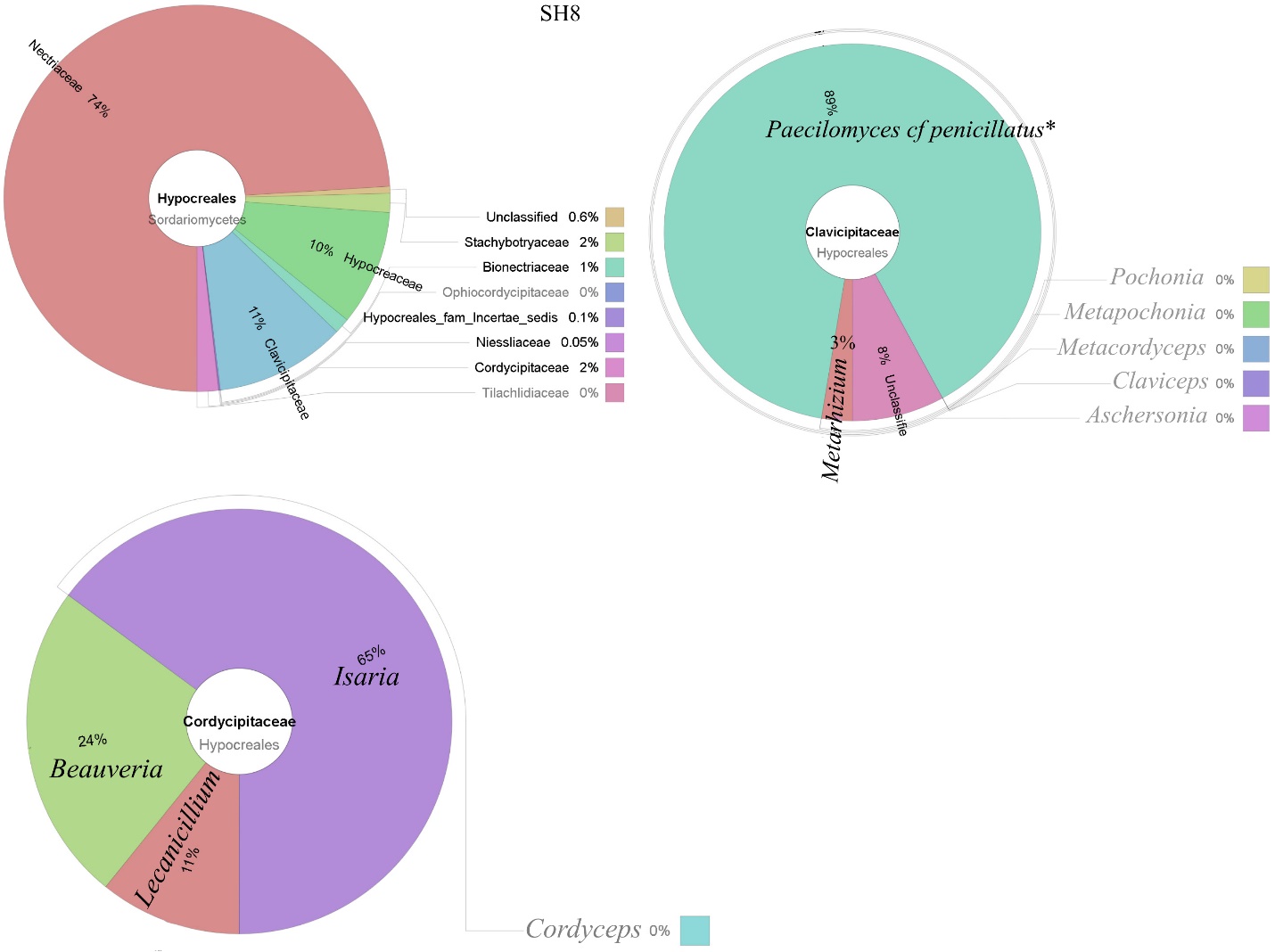


Supplementary Figure S11: Taxonomic composition of EFs in the soil samples of the one sites: SH8. * *Paecillomyces* cf *penicillatus* species does not have clear taxonomic information. Jennifer Luangsa-ard et al. (2011) showed that *P*. cf *penicillatus* was classified to Hypocreaceae family, but the information avalibility on data bases showed this species is belonged to Clavicipitaceae. However, Mycobank information (http://www.mycobank.org/name/Paecilomyces%20penicillatus&Lang=Eng) classified this genus to Trichocomaceae family. For this reason, we do not consider the frequency of this species in our main analysis.

Supplementary Figure S12: Taxonomic composition of EFs in the soil samples of the one sites: SH23. * *Paecillomyces* cf *penicillatus* species does not have clear taxonomic information. Jennifer Luangsa-ard et al. (2011) showed that *P*. cf *penicillatus* was classified to Hypocreaceae family, but the information avalibility on data bases showed this species is belonged to Clavicipitaceae. However, Mycobank information (http://www.mycobank.org/name/Paecilomyces%20penicillatus&Lang=Eng) classified this genus to Trichocomaceae family. For this reason, we do not consider the frequency of this species in our main analysis.


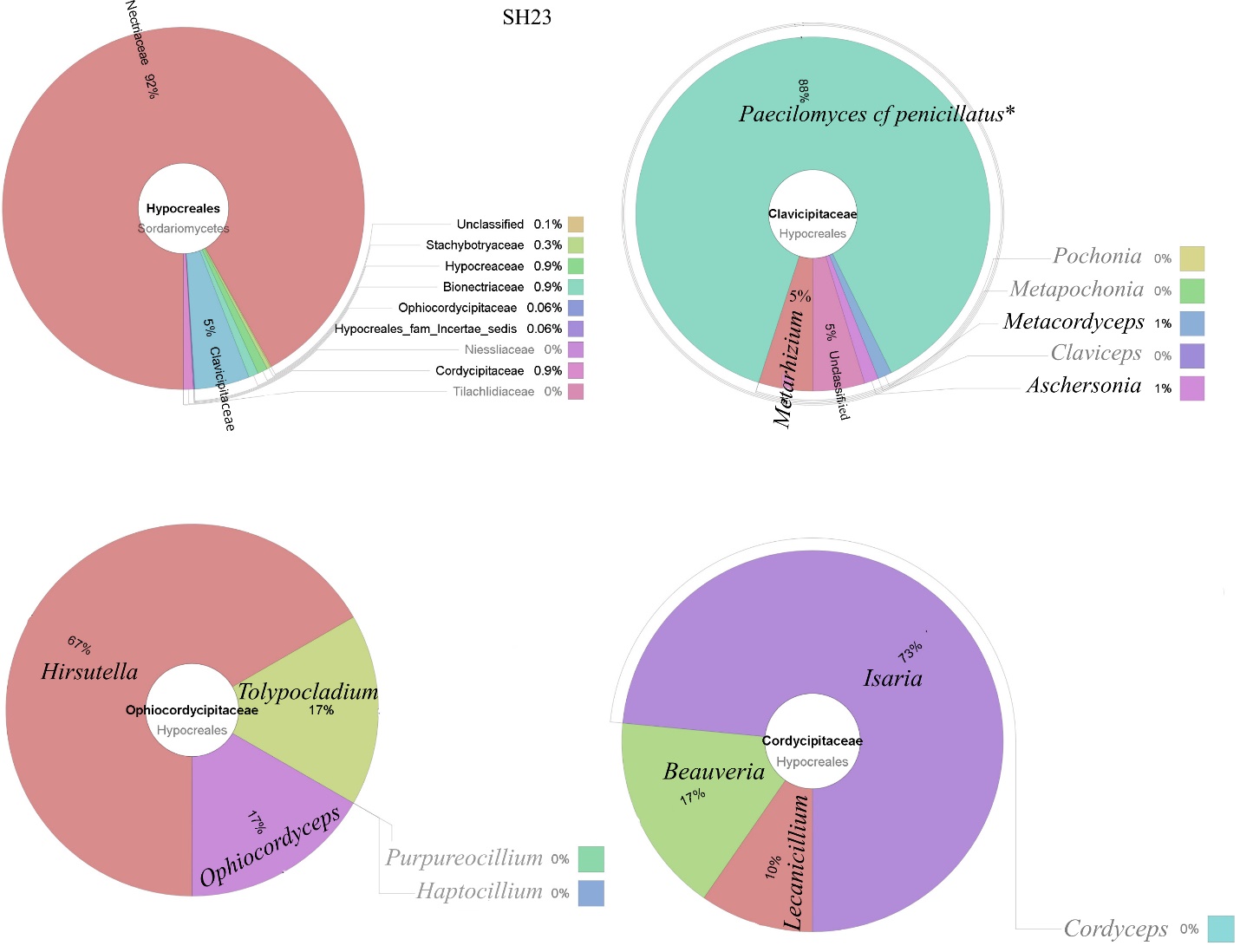


**References:**

Bischoff, J.F., Rehner, S.A., and Humber, R.A. (2009). A multilocus phylogeny of the *Metarhizium anisopliae* lineage. *Mycologia* 101**,** 512-530 DOI: 10.3852/07-202.

Kepler, R.M., Chen, Y., Kilcrease, J., Shao, J., and Rehner, S.A. (2016). Independent origins of diploidy in *Metarhizium*. *Mycologia* 108**,** 1091-1103 DOI: 10.3852/15-270.

Kepler, R.M., Humber, R.A., Bischoff, J.F., and Rehner, S.A. (2014). Clarification of generic and species boundaries for *Metarhizium* and related fungi through multigene phylogenetics. *Mycologia* 106**,** 811-829 DOI: 10.3852/13-319.

Kepler, R.M., Ugine, T.A., Maul, J.E., Cavigelli, M.A., and Rehner, S.A. (2015). Community composition and population genetics of insect pathogenic fungi in the genus *Metarhizium* from soils of a long-term agricultural research system. *Environmental Microbiology* 17**,** 2791-2804 DOI: doi:10.1111/1462-2920.12778.

Luangsa-Ard, J., Houbraken, J., Van Doorn, T., Hong, S.-B., Borman, A.M., Hywel-Jones, N.L., and Samson, R.A. (2011). *Purpureocillium*, a new genus for the medically important *Paecilomyces lilacinus*. *FEMS Microbiology Letters* 321**,** 141-149 DOI: 10.1111/j.1574-6968.2011.02322.x

Meyling, N.V., Lübeck, M., Buckley, E.P., Eilenberg, J., and Rehner, S.A. (2009). Community composition, host range and genetic structure of the fungal entomopathogen *Beauveria* in adjoining agricultural and seminatural habitats. *Molecular Ecology* 18**,** 1282-1293 DOI: <https://doi.org/10.1111/j.1365-294X.2009.04095.x>.

Rehner, S.A., and Kepler, R.M. (2017). Species limits, phylogeography and reproductive mode in the *Metarhizium anisopliae* complex. *Journal of Invertebrate Pathology* 148**,** 60-66 DOI: <https://doi.org/10.1016/j.jip.2017.05.008>.
