# Supplementary material for: Meta-Analysis and Evaluation by Insect-Mediated Baiting Reveal Different Patterns of Hypocrealean Entomopathogenic Fungi in the Soils From Two Regions of China
Source: Front Microbiol. 2020 Jun 12;11:1133. doi: 10.3389/fmicb.2020.01133 (PMC7303310; doi:10.3389/fmicb.2020.01133)
Supplement: Supplementary file 1 [file Data_Sheet_1.zip › Supplementary Tables.DOCX]

**Meta-Analysis and Evaluation by Insect-Mediated Baiting Reveal Different Patterns of Hypocrealean Entomopathogenic Fungi in the Soils From Two Regions of China**

Abolfazl Masoudi, Min Wang, Xiaoli Zhang, Can Wang, Zhaoxi Qiu, Wenying Wang, Hui Wang^*^, Jingze Liu^**^

Hebei Key Laboratory of Animal Physiology, Biochemistry and Molecular biology, College of Life Sciences, Hebei Normal University, Shijiazhuang 050024, China

Present address: 20 Nanerhuan East Road, Shijiazhuang, Hebei Province

To whom correspondence should be addressed: *Corresponding Author: Tel/fax: +86 311 80787551, [whui1981@163.com](mailto:whui1981@163.com) (H. Wang); **Corresponding Author: Tel/fax: +86 311 80787552, [liujingze@hebtu.edu.cn](mailto:liujingze@hebtu.edu.cn) (J. Liu).

Keywords: High-throughput sequencing, Entomopathogenic soil fungi, Baiting method, *Metarhizium*, *Beauveria*, Mating-type

| Supplementary Table S1: the summary of sampling locations. | | | | | | |
| --- | --- | --- | --- | --- | --- | --- |
| **Location** | **Province** | **Coordinates** | **E (m)** | **AT (ºC)** | **AP (mm)** | **HT** |
| Saihanba National Forest Park (SNFP) | Hebei | 42° 14ʹ.084ʹʹ N, 117° 08ʹ.124ʹʹ E | 1400-1600 | -1.3 | 450 | Forest and grassland |
| Taihang Shan (Taihang Mountains) | Hebei | 38° 13ʹ.406ʹʹ N, 113° 36ʹ.265ʹʹ E | 1000-2880 | 10 | 600 | Forest |
| Meishan | Sichuan | 29˚ 49´.511″ N, 102˚ 54´.272″ E | 586-955 | 16.5 | 80 | Forest |
| Ya'an | Sichuan | 29˚ 59´.953″ N, 102˚ 55´.891″ E | 592-2500 | 13.6 | 66.6 | Forest |
| Kangding to Ping Ke Xian | Sichuan to Hubei | 29˚ 49´.903″ N, 103˚ 14´.655″ E  31˚ 06´.960″ N, 113˚ 15´.184″ E | 3500-100 | 6-10 | 69-800 | Forest |
| E: Elevation  AT: Annual Temperature  AP: Annual Precipitation  HT: Habitat type | | | | | | |

| Supplemental Table S3 Overview of raw sequencing data | | | | | | | |
| --- | --- | --- | --- | --- | --- | --- | --- |
|  | **SH** | **K** | **Y** | **M** | **F** | **G** | **B** |
| Number of total reads | 76540 | 80569 | 82474 | 75801 | 76559 | 84035 | 80487 |
| Number of validate reads | 73517 | 77415 | 79150 | 73884 | 74395 | 80335 | 77670 |
| Number of bases | 22430757 | 23677902 | 24136993 | 22527159 | 22532935 | 24436401 | 23592537 |
| Average read length | 304.565 | 305.307 | 304.428 | 304.428 | 302.31 | 303.71 | 303.25 |
| Average GC % | 50.71 | 50.87 | 50.31 | 50.58 | 49.46 | 49.982 | 49.567 |
| Q20 | 83.002 | 83.146 | 84.458 | 81.502 | 84.923 | 86.107 | 85.953 |
| Effective % | 96.12 | 96.13 | 96.01 | 97.51 | 97.18 | 95.65 | 96.52 |

| Supplementary Table S4 Alpha index. EFs diversity indicated by Shannon, Simpson diversity, Chao1 richness, and Faith’s phylogenetic diversity (PD) in different sampling locations. | | | | | | | |
| --- | --- | --- | --- | --- | --- | --- | --- |
| Group | Numbers of samples | Sequence reads | Number of OTUs (Average) | Shannon | Simpson | Chao1 | PD |
| B | 8 | 405 | 14 | 1.415^ab^ | 0.58^b^ | 3.562^ab^ | 0.536 |
| F | 10 | 10121 | 32 | 1.42^ab^ | 0.56^ab^ | 4.65^ab^ | 0.562 |
| G | 10 | 459 | 14 | 0.842^a^ | 0.352^ab^ | 2.45^b^ | 0.526 |
| K | 26 | 8319 | 219 | 1.446^b^ | 0.572^ab^ | 4.135^ab^ | 0.599 |
| M | 7 | 1601 | 142 | 1.536^b^ | 0.605^a^ | 4.214^ab^ | 0.609 |
| SH | 23 | 5597 | 94 | 1.459^ab^ | 0.577^ab^ | 4.717^ab^ | 0.585 |
| Y | 14 | 5212 | 100 | 1.27^ab^ | 0.525^ab^ | 3.929^a^ | 0.518 |

| Supplementary Table S5 ANOSIM (analysis of similarity) | | |
| --- | --- | --- |
| Group | R-value | *P*-value |
| Y-K | 0.1695 | 0.003 |
| SH-K | 0.196 | 0.001 |
| SH-Y | 0.07167 | 0.086 |
| B-K | 0.009234 | 0.424 |
| B-Y | -0.03481 | 0.608 |
| B-SH | -0.02961 | 0.609 |
| F-K | 0.1752 | 0.013 |
| F-Y | -0.0021 | 0.43 |
| F-SH | 0.02854 | 0.339 |
| F-B | -0.01764 | 0.525 |
| M-K | 0.01631 | 0.403 |
| M-Y | 0.3139 | 0.004 |
| M-SH | 0.1606 | 0.056 |
| M-B | 0.3783 | 0.006 |
| M-F | 0.4864 | 0.002 |
| G-K | 0.2112 | 0.004 |
| G-Y | 0.1701 | 0.013 |
| G-SH | 0.1934 | 0.01 |
| G-B | 0.01113 | 0.324 |
| G-F | 0.05867 | 0.146 |
| G-M | 0.5528 | 0.003 |
